# Synthesis, Characterization, and Biological Evaluation of Novel 7-Oxo-7*H*-thiazolo[3,2-*b*]-1,2,4-triazine-2-carboxylic Acid Derivatives

**DOI:** 10.3390/molecules25061307

**Published:** 2020-03-13

**Authors:** Dong Cai, Tai Li, Qian Xie, Xiaofei Yu, Wei Xu, Yu Chen, Zhe Jin, Chun Hu

**Affiliations:** 1 Key Laboratory of Structure-based Drug Design & Discovery, Ministry of Education, School of Pharmaceutical Engineering, Shenyang Pharmaceutical University, Shenyang 110016, China; caidong0804@163.com (D.C.); litai159@163.com (T.L.); xqqx1996@163.com (Q.X.); yuxiaofei2017@163.com (X.Y.); 2 School of Life Science and Biopharmaceutics, Shenyang Pharmaceutical University, Shenyang 110016, China; shxuwei8720@163.com (W.X.); gzweishengwu@126.com (Y.C.)

**Keywords:** thiazolo[3,2-*b*]-1,2,4-triazinone, inhibitor, antibacterial activity, antitubercular activity, leucyl-tRNA synthetase

## Abstract

A series of novel 7-oxo-7*H*-thiazolo[3,2-*b*]-1,2,4-triazine-2-carboxylic acid derivatives was synthesized in good yields by a multi-step procedure that included the generation of the *S*-alkylated derivatives from 6-substituted arylmethyl-3-mercapto-1,2,4-triazin-5-ones with ethyl 2-chloroacetoacetate, intramolecular cyclization with microwave irradiation, hydrolysis and amidation. All of the target compounds were fully characterized through ^1^H-NMR, ^13^C-NMR and HRMS spectra. The intramolecular cyclization occurred regioselectively at the *N*2-position of 1,2,4-triazine ring, which was confirmed by compound **3e** using single-crystal X-ray diffraction analysis. The antibacterial and antitubercular activities of the target compounds were evaluated. Compared with Ciprofloxacin and Rifampicin, compounds **5d**, **5f** and **5g** containing the terminal amide fragment exhibited broad spectrum antibacterial activity, and carboxylic acid derivatives or its corresponding ethyl esters had less effect on antibacterial properties. The most potent compound **5f** also displayed excellent in vitro antitubercular activity against *Mycobacterium smegmatis* (minimum inhibitory concentration (MIC) = 50 μg/mL) and better growth inhibition activity of leucyl-tRNA synthetase (78.24 ± 4.05% at 15 μg/mL).

## 1. Introduction

Since the first discovery of penicillin by Alexander Fleming in 1928, many new antibacterial drugs have been used clinically to treat bacterial pathogen infection. However, the misuse of antibiotics has led to drug-resistant or widespread bacteria strains [1,2]. Moreover, immunocompromised patients infected with tubercular bacteria frequently also present with other severe bacterial and viral infections. Therefore, there is an urgent need for a novel multi-target agents in clinical treatment which can have an antibacterial effect and can effectively inhibit mycobacteria [3,4].

**Figure 1:**
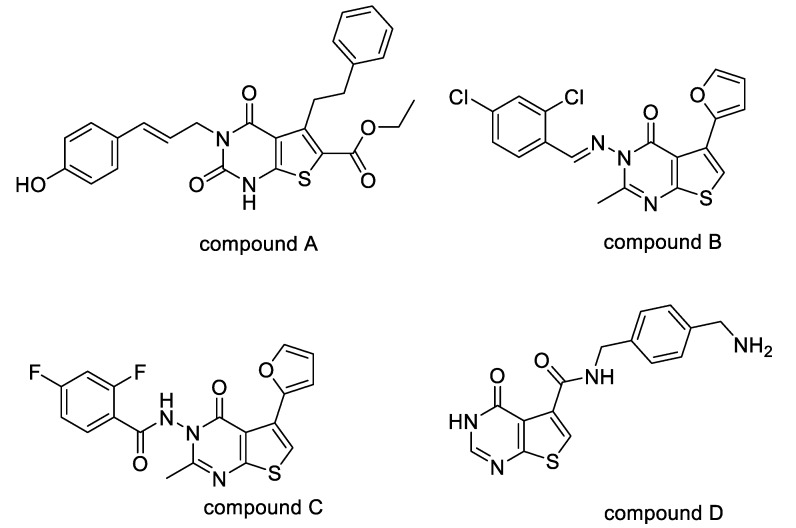
Known compounds with antimicrobial activity.

Fused thiazole derivatives have been widely used in organic and medicinal chemistry, as well as in agricultural science [5]. In a recent study, heterocycle-fused thiazole derivatives have been synthesized and investigated with the aim of finding effective antimicrobial agents [6]. In this respect, many thieno[2,3-*d*]pyrimidinones have been reported as potent antibacterial and antitubercular agents [7,8], and these scaffolds are structurally unrelated to any current clinical antibiotics. Besides this, different side chains introduced into the thieno[2,3-*d*]pyrimidinone scaffold resulted in a large difference in antibacterial activities. Compound **A** (Figure 1) possessed broad antibacterial and antimycobaterial activity, especially an anti-resistant effect [9,10]. The structure–activity relationships of this series of compounds reported in the literature demonstrated that the presence of a flexible side chain containing a phenyl ring was critical for activity. Furthermore, the presence of a 4-methoxyphenyl group increased in antibacterial effect by 2-fold when compared to the 4-hydroxyphenyl group [9,10]. The compounds containing an amido/imino linker fragment (such as **B** and **C**, Figure 1) exhibited significant antibacterial activity against tRNA-(N1G37) methyltransferase (TrmD) isoenzymes. The substituted-amido or imino side chain on thienopyrimidinone ring of compounds **B** and **C** exerted a positive influence on the antimicrobial activity [9,10]. In general, the side chains containing substituted phenyl fragments were found to be more active than the corresponding aliphatic and heterocyclic parts. Furthermore, the presence of electron-withdrawing substituents on the phenyl ring increased the potency of test compounds. The compound **D** showed most promising inhibitory activity against *Escherichia coli* with an IC_50_ of 0.91 μM (Figure 1), and the studies revealed that the hydrophobic side chain on the thiophene ring has significant effect on the antimicrobial activity [11].

Many of these studies provided a ray of hope for the emergence of a new antimicrobial leads, However, the poor bioavailability of these compounds cannot meet the medical requirements. In recent studies, optimization strategies were focused on modification of the thieno[2,3-*d*]pyrimidinone nucleus and the side chains on the nucleus.

Among the various heterocycle-fused thiazole derivatives, the thiazolo[3,2-*b*]-1,2,4-triazinone nucleus is a crucial class of *N*-bridged heterocyclic compounds with a wide range of applications in the field of therapeutic chemistry. These thiazolo[3,2-*b*]-1,2,4-triazinone derivatives have exhibited a broad spectrum of pharmacological and biological activities, such as anticancer [12], anti-inflammatory [13], antirheumatic [14] and antiacetylcholinesterase activities [15,16], etc. Comparing thieno[2,3-*d*]pyrimidinone with thiazolo[3,2-*b*]-1,2,4-triazinone nucleus using molecular overlay method, the overlay similarity value was over 60%.

**Figure 2:**
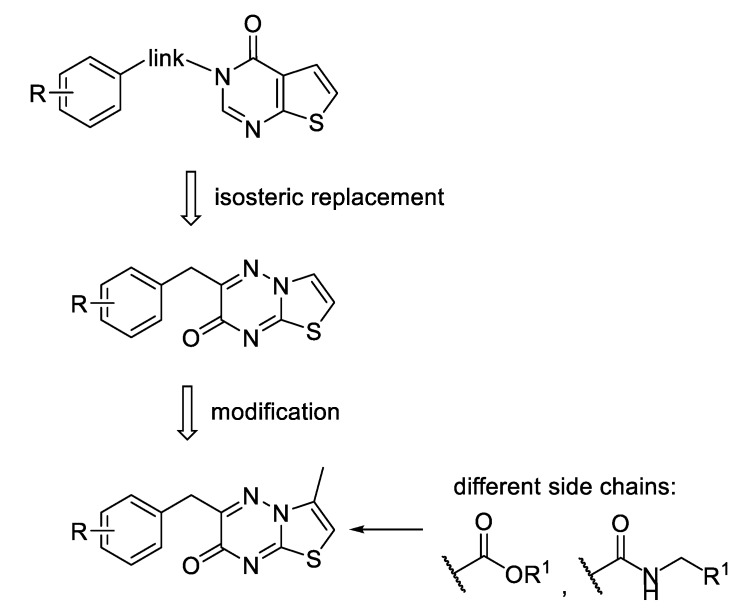
Rational design of the target compounds.

Inspired by the above-mentioned facts, a series of novel 7-oxo-7*H*-thiazolo[3,2-*b*]-1,2,4-triazine-2-carboxylic acid derivatives was designed based on the bioisosteric replacement of the thieno[2,3-*d*]pyrimidinone nucleus in potent compounds with a thiazolo[3,2-*b*]-1,2,4-triazinone nucleus. Comparing the diverse linker fragments of bioactive compounds [9,10,11], the linker part was replaced with a methylene group (-CH_2_-). As shown in Figure 2, various substituent groups were simultaneously introduced to the thiazolo[3,2-*b*]-1,2,4-triazinone nucleus to explore the structure–activity relationships.

**Figure 3:**
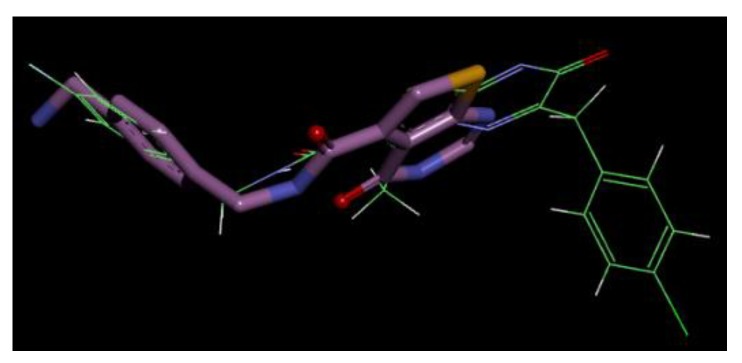
Alternative overlay of compound **D** (Sticks) and target compound **5f** (Lines). The lowest energy conformer of compound **5f** was obtained by using the full minimization calculations, and the bioactive conformer of compound **D** was produced from a crystal structure [11] (PDB code 4MCC).

The molecular overlay (Figure 3) between the compound **D** and target compound **5f** revealed a striking overlap between the bulky substituted benzyl amide side chains. Another overlapping feature was the presence of the two structural nuclei, especially the thiophene ring fragments. The structural similarity based on 50% steric field and 50% electrostatic field was accessed in the Discovery Studio 3.5 software (Accelrys Inc., San Diego, CA, USA), and the overlay similarity value was 36.15%. Thus, the structural resemblance between thieno[2,3-*d*]pyrimidinone and thiazolo[3,2-*b*]-1,2,4-triazinone indicated that the target compounds based upon the thiazolo[3,2-*b*]-1,2,4-triazinone nucleus are likely to exhibit more potent antimicrobial activities. The antibacterial and antitubercular activities of the target compounds were evaluated.

## 2. Results and Discussion

### 2.1. Chemistry

**Scheme 1:**
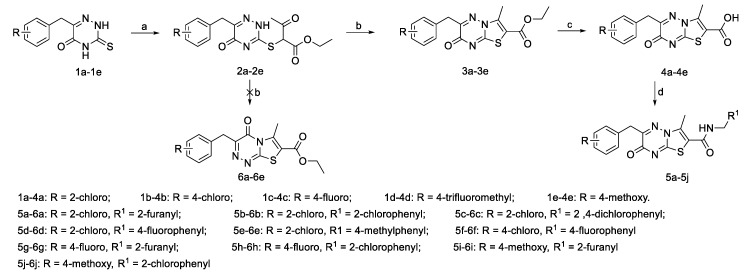
The synthetic route of the target compounds. Reagents and conditions: (a) ethyl 2-chloroacetoacetate, 10% KOH, DMF, 25 °C, 30 min; (b) polyphosphoric acid (PPA), 120 °C, 30 min, microwave; (c) 10% NaOH, CH_3_OH, 25 °C, 2 h; (d) substituted primary amine, HOBt, EDCl, triethylamine, DCM, 25 °C, 16 h.

The known approaches for the synthesis of 7-oxo-7*H*-thiazolo[3,2-*b*]-1,2,4-triazine-2-carboxylic acid derivatives can be divided into two main paths: the construction of the thiazole ring from 1,2,4-triazin-5-one derivatives [15,17] and construction of the 1,2,4-triazine ring from 2-aminothiazole derivatives [18,19,20]. There are several limitations to the second approach such as a scarcity of raw materials, low yields, and several side reactions with less selectivity of the process. Herein, a novel efficient approach was described with the aim of obtaining the target compounds in good yield and with a simple isolation procedure (Scheme 1). The synthetic approach was based on the first approach above [15]. The intermediates 6-substituted-3-mercapto-1,2,4-triazin-5-ones **1a**–**1e** were prepared using known methods [15,21]. Sulfur atoms have a larger size and lower electronegativity than those of oxygen atoms, which makes the outer electron shell more prone to donate electrons, and results in most likely nucleophilic attack [22]. The intermediates **1a**–**1e** underwent selectively rapid *S*-alkylation reaction, in which any possible *N*-, *O*-, or *C*-alkylation of the same substrate is much slower and hence negligible [23,24]. The intermediates **3a**–**3e** were synthesized by Williamson ether synthesis followed by an intramolecular cyclization reaction in PPA (polyphosphoric acid) with microwave irradiation. Next, the intermediates **3a**–**3e** underwent hydrolysis reaction in KOH methanolic solution and then were acidized to form the carboxylic acids **4a**–**4e**, which were subsequently amidated with various substituted methylamines to create the target compounds **5a**–**5j**. ^1^H-NMR and ^13^C-NMR spectra for the synthesized compounds are available in the Supplementary Materials.

Subsequently, the intermediate β-keto esters **2a**–**2e** underwent intramolecular cyclization followed by dehydration to the intermediates **3a**–**3e**. Both polyphosphoric acid (PPA) and concentrated H_2_SO_4_ were tested for their effectiveness as cyclization agents; it was found that PPA gave good results, whereas sulfuric acid made the reaction mixture a dark solution and gave several kinds of unidentified products (analyzed by TLC). This may be due to the active carbonyl group of the ethyl 2-chloroacetoacetate, resulting in the occurrence of side reactions. The reactions were studied in both classical and microwave-assisted conditions. In classical conditions, the products **3a**–**3e** were obtained in yields of 66.28%–75.26% after 60 min, while with microwave irradiation, the same products were isolated in 80.12%–87.66% yields after only 20 min (Table 1). In the cyclization with PPA, the critical factors were the reaction time and temperature. When the temperatures were above 130 °C using classical heating methods, after heating for 60 min or more, a burnt smell was dispersed. The β-keto esters **2a**–**2e** cannot be completely converted to the products below 110 °C.

**Figure 4:**
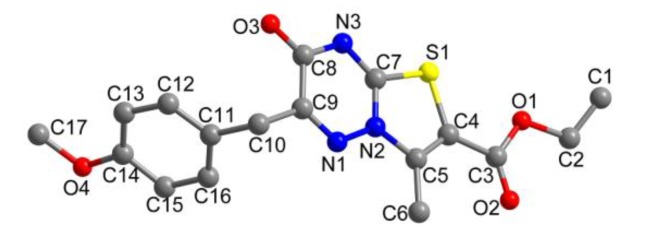
ORTEP diagram of compound **3e** at 30% Probability.

The β-keto esters **2a**–**2e** were cyclized in PPA, which yielded the intermediates **3a**–**3e**. It was reported in the literature [25] that the intramolecular condensation reaction gave the compounds **6a**–**6e** rather than the compounds **3a**–**3e** (Scheme 1). According to an X-ray structural analysis of compound **3e**, the direction of regioselective cyclization at *N*2 of the triazine ring was directly confirmed (Figure 4 and Table 2). All of the bond lengths and bond angles of compound **3e** were in the normal range. The bicyclic fragments were coplanar. The benzene ring C (11)…C (16) was almost perpendicular to the intramolecular bicyclic thiazolo[3,2-*b*]-1,2,4-triazine fragment with a *θ* angle of 89.584(90)°. In the meantime, the ester groups were nearly coplanar with the corresponding bicyclic fragment, and the torsion angles of S(1)-C(4)-C(3)-O(2) and S(1)-C(4)-C(3)-O(1) with the bicyclic were 3.278(550)° and 1.742(2185)°, respectively.

### 2.2. Biological Assays

All of the synthesized compounds were evaluated in vitro using a 96-well microtiter plate and a serial dilution method to obtain their minimum inhibitory concentration (MIC) values against two Gram-positive bacterial strains (*Staphylococcus aureus* (*S. aureus*) and *Bacillus subtilis* (*B. subtilis*)), two Gram-negative bacterial strains (*Escherichia coli* (*E. coli*), and *Pseudomonas aeruginosa* (*P. aeruginosa*)), and *Mycobacterium smegmatis* (*M. smegmatis*) [26,27]. The MIC values of these compounds are presented in Table 3.

According to the investigation of antibacterial screening data, most of the compounds exhibited low to moderate activity against Gram-positive bacteria and Gram-negative bacteria. The antibacterial activity listed in Table 3 demonstrated that, in general, the carboxylic acid derivatives **4a**–**4e** and their corresponding ethyl esters **3a**–**3e** had less effect on antibacterial properties, and compounds **3a**–**3e** containing ester moieties caused considerable losses in the inhibitory potency. Besides, the activities were only slightly affected by substituents in the 2- or 4-position of the phenyl ring of compounds **3a**–**3c** and **4a**–**4c**, which was probably attributed to a freely rotatable benzyl fragment. Furthermore, the compounds **3d** and **4d** with a *p*-substituted trifluoromethyl group at the phenyl ring had a lower antibacterial activity than the compounds **3b**, **3c**, **4b** and **4c**) with *p*-substituent halogen atoms (e.g., Cl, F). The compounds (**3e** and **4e**) were almost inactive or had low activity against all bacteria, presumably because of the presence of the electron-donating groups (e.g., CH_3_O).

Differently substituted amide chains were introduced at the carboxyl group of the thiazolo[3,2-*b*]-1,2,4-triazinone nucleus. Consequently, the antibacterial activity of compounds **5a**–**5j** was enhanced dramatically as compared with that of compounds **4a**–**4e**. It is noteworthy that the different substituents in the benzyl group on the triazine ring exert an evident influence on the antibacterial activity. The presence of the electron-withdrawing group in the phenyl ring in general increases the antibacterial activity of compounds **5a**–**5h** compared to compounds **5i**–**5j** containing electron-donating groups. In the meantime, the comparison of the compounds **5a**–**5j** with the different terminal amide fragment indicated that there was no significant difference in activity.

Obviously, only one compound **5f** exhibited significant antitubercular activity against *M. smegmatis*, compared with the reference compound of Rifampicin.

### 2.3. Target Identification by Pharmacophore

Automated pharmacophoric profiling is a readily available and effective target fishing method which is conducted by using the ligand profiler protocol, which can be used to explore the most likely targets of a query chemical structure or determine the off-targets of an existing drug [26,27]. To provide more evidence for the possible binding targets of the synthesized compounds, the compound **5f** was selected to evaluate the antibacterial and antitubercular properties on a molecular level. The FitValue was used as an important judgment of the overlap degree between the query compound and pharmacophore models [28].

According to the screening results (Table 4), compound **5f** was able to map some effective antimicrobial pharmacophore models, including methyltransferases, aminoacyl-tRNA synthetases (AARSs), and enoyl-[acyl-carrier-protein] reductase (NADH). The results indicated that compound **5f** might have good antimicrobial activity, especially inhibiting bacterial thymidylate synthase and AARSs. In practice, the bacterial thymidylate synthase is a highly conserved protein in bacteria and humans, and it has proven to be unsuitable as a target for selective antibacterial inhibitors [29]. The AARSs family are key components in protein synthesis; among them, leucyl-tRNA synthetase (LeuRS) has been validated as a promising target for antimicrobial drug therapy. Based on the above results, compound **5f** could be regarded as a potential LeuRS inhibitor candidate for further research.

### 2.4. Leucyl-tRNA Synthetase Activity

The enzyme inhibition data against LeuRS from *M. smegmatis* for the selected compounds are given in Table 5. It was found that compound **5f** with a 4-fluorophenyl group at R_1_ was more potent than its carboxylic acid precursor **4b**. Compound **5f** exhibited a high inhibitory effect on the *M. smegmatis* LeuRS with the percent inhibition of 78.24 ± 4.05% at 15 μg/mL, which is a six-fold improvement as compared to compound **4b** (with a percentage inhibition of 12.89 ± 2.31% at 15 μg/mL). The obtained results showed that novel 7-oxo-7*H*-thiazolo[3,2-*b*]-1,2,4-triazine-2-carboxylic acid derivatives seem to be effective *M. smegmatis* LeuRS inhibitors and worthy of further exploitation.

## 3. Materials and Methods

### 3.1. General Information

All solvents and chemicals were purchased from common commercial suppliers and were used without further purification. Melting points were taken in open capillary tubes and are reported uncorrected. IR spectra were recorded on a FT-IR 920 spectrophotometer (Tianjin Tuopu Instrument Co., Ltd., Tianjin, China) using KBr pellets. The ^1^H- and ^13^C-NMR spectra were obtained with TMS as internal standard using a 400/54 Premium Shielded NMR Magnet System (Agilent Technologies, Santa Clara, CA, USA). Mass spectra were determined on an Agilent 6200 Series TOF and 6500 Series Q-TOF LC/MS System B.05.01. (B5125, Agilent Technologies). The microwave assisted reactions were carried out in a synthetic microwave apparatus (Shanghai Biaohe Instrument Co., Ltd., Shanghai, China). X-ray single-crystal structure determinations were carried out on a Bruker SMART APEX II CCD diffractometer (Bruker AXS GMBH, Karlsruhe, Germany).

The original figures of IR, ^1^H-NMR, ^13^C-NMR, MS, and HRMS of all the target compounds and the key intermediates are available online.

### 3.2. Synthesis

#### 3.2.1. General Procedure for the Synthesis of the β-Keto Esters (**2a**–**2e**)

A 10% aqueous solution of KOH (5.6 mL) was added dropwise under stirring to form a suspension of compound **1** (10 mmol) in DMF (10 mL). Then, ethyl 2-chloroacetoacetate (1.4 mL, 10 mmol) was added and the mixture was stirred at 25 °C for 30 min. Then, the mixture was poured into ice water to obtain compound **2**, which was filtered and washed with water, and then subsequently recrystallized from ethyl acetate.

*Ethyl 2-((6-(2-chlorobenzyl)-5-oxo-2,5-dihydro-1,2,4-triazin-3-yl)thio)-3-oxobutanoate* (**2a**): White solid; Yield: 73.12%; mp: 129.4–130.1 °C; IR (KBr): υ 3260, 2986, 2950, 1743, 1653, 1575, 1482, 1422, 1309, 1184, 1018, 750, 686 cm^−1^; ^1^H-NMR (400 MHz, DMSO-*d*_6_) δ 7.79 (s, 1H), 7.46 (dt, *J* = 7.6, 2.9 Hz, 1H), 7.39–7.24 (m, 3H), 5.16 (s, 1H), 4.21 (qd, *J* = 7.1, 3.0 Hz, 2H), 4.09–3.94 (m, 2H), 1.79 (s, 3H), 1.24 (t, *J* = 7.1 Hz, 3H); HRMS (*m*/*z*): calcd. for C_16_H_17_ClN_3_O_4_S (M + H^+^) 382.06283, found 382.06076.

*Ethyl 2-((6-(4-chlorobenzyl)-5-oxo-2,5-dihydro-1,2,4-triazin-3-yl)thio)-3-oxobutanoate* (**2b**): White solid; Yield: 75.34%; mp: 126.8–128.7 °C; IR (KBr): υ 3392, 3100, 2982, 2933, 1721, 1654, 1580, 1501, 1407, 1316, 1273, 1203, 806 cm^−1^; ^1^H-NMR (400 MHz, DMSO-*d*_6_) δ 7.85 (s, 1H), 7.42–7.32 (m, 2H), 7.33–7.17 (m, 2H), 5.15 (s, 1H), 4.22 (tdd, *J* = 7.2, 5.5, 2.5 Hz, 2H), 3.93–3.75 (m, 2H), 1.89 (s, 3H), 1.26 (d, *J* = 7.1 Hz, 3H); HRMS (*m*/*z*): calcd. for C_16_H_17_ClN_3_O_4_S (M + H^+^) 382.06283, found 382.06247.

*Ethyl 2-((6-(4-fluorobenzyl)-5-oxo-2,5-dihydro-1,2,4-triazin-3-yl)thio)-3-oxobutanoate* (**2c**): White solid; Yield: 72.69%; mp: 125.1–125.4 °C; IR (KBr): υ 3388, 2987, 2930, 1724, 1659, 1578, 1505, 1410, 1316, 1210, 1021, 810 cm^−1^; ^1^H-NMR (400 MHz, DMSO-*d*_6_) δ 7.88 (d, *J* = 33.9 Hz, 1H), 7.36–7.25 (m, 2H), 7.18–7.09 (m, 2H), 5.15 (s, 1H), 4.22 (qd, *J* = 7.1, 2.0 Hz, 2H), 3.94–3.79 (m, 2H), 1.89 (s, 3H), 1.25 (t, *J* = 7.1 Hz, 3H); HRMS (*m*/*z*): calcd. for C_16_H_17_FN_3_O_4_S (M + H^+^) 366.09238, found 366.09278.

*Ethyl 3-oxo-2-((5-oxo-6-(4-(trifluoromethyl)benzyl)-2,5-dihydro-1,2,4-triazin-3-yl)thio)butanoate* (**2d**): White solid; Yield: 76.52%; mp: 128.1–128.7 °C; IR (KBr): υ 3121, 2997, 1741, 1633, 1580, 1501, 1434, 1368, 1263, 1170, 1028, 856 cm^−1^; ^1^H-NMR (400 MHz, DMSO-*d*_6_) δ 7.84 (s, 1H), 7.44–7.36 (m, 2H), 7.31 (d, *J* = 8.2 Hz, 2H), 5.15 (s, 1H), 4.22 (qd, *J* = 7.1, 2.1 Hz, 2H), 3.99–3.84 (m, 2H), 1.89 (s, 3H), 1.25 (t, *J* = 7.1 Hz, 3H); HRMS (*m*/*z*): calcd. for C_17_H_20_F_3_N_4_O_4_S (M + NH_4_^+^) 433.11574, found 433.08624.

*Ethyl 2-((6-(4-methoxybenzyl)-5-oxo-2,5-dihydro-1,2,4-triazin-3-yl)thio)-3-oxobutanoate* (**2e**): Yellow solid; Yield: 80.38%; mp: 131.2–132.6 °C; IR (KBr): υ 3215, 2986, 2947, 1731, 1630, 1573, 1494, 1424, 1304, 1247, 1179, 1095, 820 cm^−1^; ^1^H-NMR (400 MHz, DMSO-*d*_6_) δ 7.85 (s, 1H), 7.24–7.15 (m, 2H), 6.90–6.81 (m, 2H), 5.15 (s, 1H), 4.22 (qd, *J* = 7.1, 2.0 Hz, 2H), 3.87–3.73 (m, 2H), 3.72 (s, 3H), 1.91 (s, 3H), 1.25 (t, *J* = 7.2 Hz, 3H); HRMS (*m*/*z*): calcd. for C_17_H_20_N_3_O_5_S (M + H^+^) 378.11237, found 378.10079.

#### 3.2.2. General Procedure for the Synthesis of Ethyl 6-Aryl-3-methyl-7-oxo-7*H*-thiazolo[3,2-*b*]-1,2,4-triazine-2-carboxylates (**3a**–**3e**)

Compound **2** (0.01 mol) was added to PPA (85% P_2_O_5_) (10.0 g). The mixture was irradiated in a microwave at 120 °C for 20 min at a power of 400 W. The reaction mixture was then poured into ice water. The resulting solid was filtered, washed with cold water (3 × 20 mL) and dried. The crude product was recrystallized from ethyl acetate to give compound **3**.

*Ethyl 6-(2-chlorobenzyl)-3-methyl-7-oxo-7H-thiazolo[3,2-b]-1,2,4-triazine-2-carboxylate* (**3a**): White solid; Yield: 81.24%; mp: 162.3–163.3 °C; IR (KBr): υ 3059, 2986, 2941, 1697, 1609, 1495, 1474, 1242, 1196, 760 cm^−1^; ^1^H-NMR (400 MHz, Chloroform-*d*) δ 7.41 (ddd, *J* = 8.7, 5.4, 3.5 Hz, 2H), 7.26 (dd, *J* = 5.8, 3.5 Hz, 2H), 4.40 (q, *J* = 7.1 Hz, 2H), 4.33 (s, 2H), 2.60 (s, 3H), 1.40 (t, *J* = 7.1 Hz, 3H); ^13^C-NMR (101 MHz, Chloroform-*d*) δ 163.94, 160.49, 158.92, 154.33, 143.13, 134.72, 133.15, 131.81, 129.45, 128.65, 126.85, 109.99, 62.43, 35.14, 14.17, 11.86; HRMS (*m*/*z*): calcd. for C_16_H_1__5_ClN_3_O_3_S (M + H^+^) 364.05226, found 364.05240.

*Ethyl 6-(4-chlorobenzyl)-3-methyl-7-oxo-7H-thiazolo[3,2-b]-1,2,4-triazine-2-carboxylate* (**3b**): White solid; Yield: 85.12%; mp: 130.7–132.1 °C; IR (KBr): υ 3030, 2984, 2960, 1715, 1585, 1501, 1463, 1285, 1221, 808 cm^−1^; ^1^H-NMR (600 MHz, DMSO-*d*_6_) δ 7.35 (s, 4H), 4.33 (q, *J* = 7.1 Hz, 2H), 4.01 (s, 2H), 2.61 (s, 3H), 1.30 (t, *J* = 7.1 Hz, 3H); ^13^C-NMR (101 MHz, Chloroform-*d*) δ 164.05, 160.45, 158.82, 155.18, 142.98, 133.54, 133.04, 130.93, 128.66, 110.28, 62.50, 36.61, 14.18, 12.08; HRMS (*m*/*z*): calcd. for C_16_H_1__5_ClN_3_O_3_S (M + H^+^) 364.05226, found 364.05270.

*Ethyl 6-(4-fluorobenzyl)-3-methyl-7-oxo-7H-thiazolo[3,2-b]-1,2,4-triazine-2-carboxylate* (**3c**): White solid; Yield: 83.78%; mp: 128.3–129.1 °C; IR (KBr): υ 3066, 2984,2938, 1715,1501,1463, 1285, 1221, 808 cm^−1^; ^1^H-NMR (400 MHz, Chloroform-*d*) δ 7.39 (dd, *J* = 8.6, 5.4 Hz, 2H), 7.02 (t, *J* = 8.7 Hz, 2H), 4.42 (q, *J* = 7.1 Hz, 2H), 4.13 (s, 2H), 2.76 (s, 3H), 1.41 (t, *J* = 7.1 Hz, 3H); ^13^C-NMR (101 MHz, Chloroform-*d*) δ 164.03, 163.21, 160.77, 160.48, 158.84, 155.42, 142.99, 131.18, 131.10, 115.49, 115.28, 110.20, 62.48, 36.46, 14.17, 12.06; HRMS (*m*/*z*): calcd. for C_16_H_15_FN_3_O_3_S (M + H^+^) 348.08182, found 348.08240.

*Ethyl 3-methyl-7-oxo-6-(4-(trifluoromethyl)benzyl)-7H-thiazolo[3,2-b]-1,2,4-triazine-2-carboxylate* (**3d**): White solid; Yield: 80.12%; mp: 94.9–95.2 °C; IR (KBr): υ 2998, 2918, 1720, 1490, 1267, 1161, 816 cm^−1^; ^1^H-NMR (400 MHz, Chloroform-*d*) δ 7.45 (d, *J* = 8.5 Hz, 2H), 7.18 (d, *J* = 8.0 Hz, 2H), 4.42 (q, *J* = 7.1 Hz, 2H), 4.16 (s, 2H), 2.77 (s, 3H), 1.42 (t, *J* = 7.1 Hz, 3H); ^13^C-NMR (101 MHz, Chloroform-*d*) δ 165.36, 158.74, 156.67, 156.37, 154.83, 152.49, 148.11, 146.62, 130.59, 120.73, 109.93, 62.23, 36.25, 13.85, 11.79; HRMS (*m*/*z*): calcd. for C_17_H_18_F_3_N_4_O_3_S (M + NH_4_^+^) 415.10517, found 415.07679.

*Ethyl 6-(4-methoxybenzyl)-3-methyl-7-oxo-7H-thiazolo[3,2-b]-1,2,4-triazine-2-carboxylate* (**3e**): Yellow solid; Yield: 87.66%; mp: 105.3–105.5 °C; IR (KBr): υ 2982, 2918, 1721, 1695, 1659, 1613, 1575, 1505, 1368, 1290, 1242, 1174, 1131, 1052, 1028, 815 cm^−1^; ^1^H-NMR (400 MHz, Chloroform-*d*) δ 7.38–7.30 (m, 2H), 6.91–6.82 (m, 2H), 4.41 (q, *J* = 7.1 Hz, 2H), 4.10 (s, 2H), 3.81 (s, 3H), 2.77 (s, 3H), 1.42 (d, *J* = 7.1 Hz, 3H); ^13^C-NMR (101 MHz, Chloroform-*d*) δ 163.95, 160.54, 158.94, 158.67, 155.75, 143.08, 130.61, 127.04, 113.94, 109.98, 62.43, 55.24, 36.35, 14.18, 12.09; HRMS (*m*/*z*): calcd. for C_17_H_18_N_3_O_4_S (M + H^+^) 360.10180, found 360.09732.

#### 3.2.3. General Procedure for the Synthesis of 6-Aryl-3-methyl-7-oxo-7H-thiazolo[3,2-*b*]-1,2,4-triazine-2-carboxylic acids (**4a**–**4e**)

The ester derivative (**3**) (4.0 mmol) was hydrolyzed by treatment with a mixture of NaOH 10% (10 mL) and CH_3_OH (10 mL) for 2 h at 25 °C. The solution was concentrated to half volume and the product was precipitated by acidification with 10% HCl to pH value of 3.0, filtered, washed with water, then dried.

*6-(2-Chlorobenzyl)-3-methyl-7-oxo-7H-thiazolo[3,2-b]-1,2,4-triazine-2-carboxylic acid* (**4a**): White solid; Yield: 95.35%; mp: 209.6–210.4 °C; IR (KBr): υ 2920, 1629,1577, 1475, 1440, 1351, 1038, 751 cm^−1^; ^1^H-NMR (600 MHz, DMSO-*d*_6_) δ 7.46 (dd, *J* = 7.5, 1.8 Hz, 1H), 7.42–7.37 (m, 1H), 7.31–7.26 (m, 2H), 4.13 (s, 2H), 2.46 (s, 3H); ^13^C-NMR (101 MHz, DMSO-*d*_6_) δ 164.01, 162.63, 158.99, 153.55, 134.20, 133.96, 131.91, 129.55, 129.11, 127.48, 34.79, 11.68; HRMS (*m*/*z*): calcd. for C_14_H_11_ClN_3_O_3_S (M + H^+^) 336.02096, found 336.02300.

*6-(4-Chlorobenzyl)-3-methyl-7-oxo-7H-thiazolo[3,2-b]-1,2,4-triazine-2-carboxylic acid* (**4b**): White solid; Yield: 93.11%; mp: 197.2–198.2 °C; IR (KBr): υ 2959, 2927, 1717, 1649, 1613, 1575, 1484, 1417, 1356, 1242, 1093, 803 cm^−1^; ^1^H-NMR (400 MHz, DMSO-*d*_6_) δ 7.37 (s, 4H), 4.02 (s, 2H), 2.61 (s, 3H); ^13^C-NMR (101 MHz, DMSO-*d*_6_) δ 164.10, 162.66, 159.00, 154.26, 142.19, 135.29, 131.78, 131.61, 131.10, 128.60, 36.51, 12.04; HRMS (*m*/*z*): calcd. for C_14_H_11_ClN_3_O_3_S (M + H^+^) 336.02096, found 336.02190.

*6-(4-Fluorobenzyl)-3-methyl-7-oxo-7H-thiazolo[3,2-b]-1,2,4-triazine-2-carboxylic acid* (**4c**): White solid; Yield: 91.34%; mp: 205.9–206.2 °C; IR (KBr): υ 2950, 1697, 1569, 1507, 1470, 1287, 1252, 1015, 809 cm^−1^; ^1^H-NMR (600 MHz, DMSO-*d*_6_) δ 7.36 (dd, *J* = 8.4, 5.5 Hz, 2H), 7.12 (t, *J* = 8.8 Hz, 2H), 3.99 (s, 2H), 2.59 (s, 3H); ^13^C-NMR (101 MHz, DMSO-*d*_6_) δ 164.10, 162.76, 162.61, 160.35, 159.02, 154.49, 132.33, 132.30, 131.67, 131.59, 115.52, 115.30, 36.34, 12.07; HRMS (*m*/*z*): calcd. for C_14_H_11_FN_3_O_3_S (M + H^+^) 320.05052, found 320.05222.

*3-Methyl-7-oxo-6-(4-(trifluoromethyl)benzyl)-7H-thiazolo[3,2-b]-1,2,4-triazine-2-carboxylic acid* (**4d**): White solid; Yield: 89.88%; mp: 212.6–213.8 °C; IR (KBr): υ 3049, 2957, 1711, 1612, 1508, 1476, 1423, 1381, 1354, 1286, 921, 779, 764 cm^−1^; ^1^H-NMR (600 MHz, DMSO-*d*_6_) δ 7.46 (d, *J* = 8.2 Hz, 2H), 7.29 (d, *J* = 8.1 Hz, 2H), 4.04 (s, 2H), 2.59 (s, 3H); ^13^C-NMR (101 MHz, DMSO-*d*_6_) δ 164.15, 162.64, 159.01, 154.23, 147.57, 142.45, 135.81, 131.59, 121.77, 121.32, 119.23, 36.50, 12.05; HRMS (*m*/*z*): calcd. for C_15_H_1__1_F_3_N_3_O_3_S (M + NH_4_^+^) 387.07387, found 387.04752.

*6-(4-Methoxybenzyl)-3-methyl-7-oxo-7H-thiazolo[3,2-b]-1,2,4-triazine-2-carboxylic acid* (**4e**): Yellow solid; Yield: 94.50%; mp: 209.8–210.8 °C; IR (KBr): υ 2944, 2835, 2752, 2595, 2503, 1709, 1662, 1618, 1580, 1486, 1434, 1359, 1273, 1244, 1184, 1026, 767 cm^−1^; ^1^H-NMR (400 MHz, DMSO-*d*_6_) δ 14.27 (s, 1H), 7.30–7.21 (m, 2H), 6.91–6.82 (m, 2H), 3.94 (s, 2H), 3.72 (s, 3H), 2.63 (s, 3H); ^13^C-NMR (101 MHz, DMSO-*d*_6_) δ 164.05, 162.64, 158.98, 158.49, 154.80, 142.91, 130.77, 127.88, 114.12, 110.63, 55.44, 36.29, 12.17; HRMS (*m*/*z*): calcd. for C_15_H_1__4_N_3_O_4_S (M + H^+^) 332.07050, found 332.07303.

#### 3.2.4. General Procedure for the Synthesis of the Target Compounds **5a**–**5j**

Substituted primary amine (1.0 mmol) was added to a solution of compound **4** (1.0 mmol), HOBt (1-hydroxybenzotriazole, 0.15 g, 1.1 mmol), triethylamine (0.34 g, 3.3 mmol), and EDCl (*N*′-(3-dimethylaminopropyl)-*N*-ethylcarbodiimide hydrochloride, 0.21 g, 1.1 mmol) in DCM (dichloromethane, 30 mL). The solution was then stirred at 25 °C for 16 h. The reaction was diluted with H_2_O (30 mL), and the organic layer was separated and sequentially washed with 1 mol·L^−1^ HCl (10 mL), saturated NaHCO_3_ aqueous solution (10 mL), and brine (10 mL), dried (Na_2_SO_4_). The solvent was filtrated and evaporated, resulting in the crude product which was purified by chromatography (CH_2_Cl_2_-CH_3_OH, V:V = 50:1).

*6-(2-Chlorobenzyl)-N-(furan-2-ylmethyl)-3-methyl-7-oxo-7H-thiazolo[3,2-b]-1,2,4-triazine-2-carboxamide* (**5a**): White solid; Yield: 72.60%; mp: 138.5–139.0 °C; IR (KBr): υ 3331, 3055, 2926, 1655, 1609, 1570, 1487, 1443, 1344, 748 cm^−1^; ^1^H-NMR (400 MHz, Chloroform-*d*) δ 7.40 (qd, *J* = 5.7, 3.5 Hz, 3H), 7.25 (dd, *J* = 5.8, 3.5 Hz, 2H), 6.41–6.30 (m, 2H), 6.06 (s, 1H), 4.61 (d, *J* = 5.1 Hz, 2H), 4.32 (s, 2H), 2.56 (s, 3H); ^13^C-NMR (101 MHz, Chloroform-*d*) δ 162.97, 159.67, 159.13, 154.08, 150.16, 142.44, 139.14, 134.65, 133.09, 131.74, 129.45, 129.40, 128.66, 126.84, 113.35, 110.53, 108.17, 37.07, 35.12, 12.11; HRMS (*m*/*z*): calcd. for C_19_H_16_ClN_4_O_3_S (M + H^+^) 415.06316, found 415.06469.

*N,6-Bis(2-chlorobenzyl)-3-methyl-7-oxo-7H-thiazolo[3,2-b]-1,2,4-triazine-2-carboxamide* (**5b**): White solid; Yield: 72.20%; mp: 148.1–149.4 °C; IR (KBr): υ 3271, 3073, 2940, 1663, 1614, 1575, 1504, 1440, 1352, 752 cm^–1^; ^1^H-NMR (400 MHz, Chloroform-*d*) δ 7.48–7.36 (m, 4H), 7.32–7.20 (m, 4H), 6.35 (s, 1H), 4.69 (d, *J* = 5.4 Hz, 2H), 4.31 (s, 2H), 2.54 (s, 3H); ^13^C-NMR (101 MHz, Chloroform-*d*) δ 163.00, 159.79, 159.04, 154.06, 138.74, 134.66, 134.57, 133.57, 133.11, 131.74, 130.37, 129.62, 129.45, 129.31, 128.66, 127.17, 126.84, 113.68, 42.34, 35.11, 12.11; HRMS (*m*/*z*): calcd. for C_21_H_17_Cl_2_N_4_O_2_S (M + H^+^) 459.04493, found 459.06700.

*6-(2-Chlorobenzyl)-N-(2,4-dichlorobenzyl)-3-methyl-7-oxo-7H-thiazolo[3,2-b]-1,2,4-triazine-2-carboxamide* (**5c**): White solid; Yield: 71.40%; mp: 209.8–252.4 ^°^C; IR (KBr): υ 3254, 3062, 2928, 1638, 1616, 1570, 1541, 1487, 1441, 1387, 756 cm^–1^; ^1^H-NMR (400 MHz, Chloroform-*d*) δ 7.46–7.32 (m, 4H), 7.30–7.20 (m, 3H), 6.48 (d, *J* = 6.1 Hz, 1H), 4.65 (d, *J* = 5.9 Hz, 2H), 4.30 (s, 2H), 2.54 (s, 3H); ^13^C-NMR (101 MHz, Chloroform-*d*) δ 162.90, 159.75, 158.96, 154.20, 139.15, 134.70, 134.65, 134.30, 133.10, 131.78, 131.50, 129.54, 129.47, 128.68, 127.54, 126.86, 112.96, 41.88, 35.15, 12.10; HRMS (*m*/*z*): calcd. for C_21_H_16_Cl_3_N_4_O_2_S (M + H^+^) 493.00595, found 493.0835.

*6-(2-Chlorobenzyl)-N-(4-fluorobenzyl)-3-methyl-7-oxo-7H-thiazolo[3,2-b]-1,2,4-triazine-2-carboxamide* (**5d**): White solid; Yield: 69.90%; mp: 153.5–154.4 °C; IR (KBr): υ 3059, 2922, 2851, 1640, 1578, 1509, 1480, 1434, 1385, 765 cm^–1^; ^1^H-NMR (400 MHz, Chloroform-*d*) δ 7.42–7.24 (m, 6H), 7.08–7.03 (m, 2H), 6.34 (s, 1H), 4.57 (d, *J* = 5.6 Hz, 2H), 4.29 (s, 2H), 2.55 (s, 3H); ^13^C-NMR (101 MHz, Chloroform-*d*) δ 163.38, 162.90, 160.93, 159.82, 159.18, 154.02, 139.13, 134.61, 133.32, 133.28, 133.03, 131.67, 129.73, 129.65, 129.47, 128.69, 126.85, 115.61, 115.40, 113.53, 43.52, 35.10, 12.13; HRMS (*m*/*z*): calcd. for C_21_H_17_ClFN_4_O_2_S (M + H^+^) 443.07448, found 443.07726.

*6-(2-Chlorobenzyl)-3-methyl-N-(4-methylbenzyl)-7-oxo-7H-thiazolo[3,2-b]-1,2,4-triazine-2-carboxamide* (**5e**): White solid; Yield: 72.00%; mp: 166.6–167.3 °C; IR (KBr): υ 2920, 2851, 1654, 1569, 1532, 1476, 1440, 1384, 764 cm^–1^; ^1^H-NMR (400 MHz, Chloroform-*d*) δ 7.39 (ddd, *J* = 11.2, 5.5, 3.5 Hz, 2H), 7.30–7.15 (m, 6H), 6.09 (s, 1H), 4.56 (d, *J* = 5.3 Hz, 2H), 4.30 (s, 2H), 2.55 (s, 3H), 2.36 (s, 3H); ^13^C-NMR (101 MHz, Chloroform-*d*) δ 162.85, 159.64, 159.06, 154.07, 139.02, 137.69, 134.68, 134.03, 133.13, 131.76, 129.48, 129.46, 128.66, 127.93, 126.84, 113.25, 44.13, 35.11, 21.10, 12.09; HRMS (*m*/*z*): calcd. for C_22_H_20_ClN_4_O_2_S (M + H^+^) 439.09955, found 439.10209.

*6-(4-Chlorobenzyl)-N-(4-fluorobenzyl)-3-methyl-7-oxo-7H-thiazolo[3,2-b]-1,2,4-triazine-2-carboxamide* (**5f**): White solid; Yield: 70.20%; mp: 210.8–211.2 °C; IR (KBr): υ 3246, 3076, 2939, 1652, 1607, 1570, 1532, 1508, 1373, 1352, 836, 807 cm^–1^; ^1^H-NMR (400 MHz, Chloroform-*d*) δ 7.39–7.25 (m, 6H), 7.07 (t, *J* = 8.6 Hz, 2H), 6.18 (s, 1H), 4.59 (d, *J* = 5.7 Hz, 2H), 4.10 (s, 2H), 2.72 (s, 3H); ^13^C-NMR (101 MHz, Chloroform-*d*) δ 162.83, 159.62, 158.86, 155.06, 139.42, 133.46, 133.10, 130.88, 129.78, 129.70, 128.69, 115.91, 115.69, 112.72, 43.65, 36.61, 12.33; HRMS (*m*/*z*): calcd. for C_21_H_17_ClFN_4_O_2_S (M + H^+^) 443.07448, found 443.07610.

*6-(4-Fluorobenzyl)-N-(furan-2-ylmethyl)-3-methyl-7-oxo-7H-thiazolo[3,2-b]-1,2,4-triazine-2-carboxamide* (**5g**): White solid; Yield: 73.30%; mp: 155.4–155.8 °C; IR (KBr): υ 3231, 3126, 3063, 2932, 1636, 1568, 1508, 1479, 1418, 1346, 812 cm^–1^; ^1^H-NMR (400 MHz, Chloroform-*d*) δ 7.39 (dd, *J* = 1.8, 0.8 Hz, 1H), 7.38–7.32 (m, 2H), 7.05–6.94 (m, 2H), 6.42–6.28 (m, 3H), 4.61 (d, *J* = 5.4 Hz, 2H), 4.10 (s, 2H), 2.71 (s, 3H); ^13^C-NMR (101 MHz, Chloroform-*d*) δ 163.15, 163.05, 160.71, 159.68, 159.08, 155.09, 150.27, 142.38, 139.06, 131.11, 131.03, 130.72, 130.69, 115.44, 115.22, 113.46, 110.52, 108.15, 37.04, 36.44, 12.31; HRMS (*m*/*z*): calcd. for C_19_H_16_FN_4_O_3_S (M + H^+^) 399.09271, found 399.09391.

*N-(2-Chlorobenzyl)-6-(4-fluorobenzyl)-3-methyl-7-oxo-7H-thiazolo[3,2-b]-1,2,4-triazine-2-carboxamide* (**5h**): White solid; Yield: 69.40%; mp: 161.7–162.9 °C; IR (KBr): υ 3331, 3057, 2937, 1639, 1616, 1572, 1510, 1477, 1441, 1348, 804, 752 cm^−1^; ^1^H-NMR (400 MHz, Chloroform-*d*) δ 7.50–7.27 (m, 6H), 7.01 (t, *J* = 8.7 Hz, 2H), 6.27 (s, 1H), 4.71 (d, *J* = 5.9 Hz, 2H), 4.11 (s, 2H), 2.71 (s, 3H); ^13^C-NMR (101 MHz, Chloroform-*d*) δ 163.16, 163.07, 160.71, 159.81, 159.01, 155.07, 138.76, 134.62, 133.52, 131.09, 131.01, 130.72, 130.69, 130.21, 129.59, 129.22, 127.12, 115.45, 115.24, 113.70, 42.28, 36.42, 12.29; HRMS (*m*/*z*): calcd. for C_21_H_17_ClFN_4_O_2_S (M + H^+^) 443.07448, found 443.07588.

*N-(Furan-2-ylmethyl)-6-(4-methoxybenzyl)-3-methyl-7-oxo-7H-thiazolo[3,2-b]-1,2,4-triazine-2-carboxamide* (**5i**): White solid; Yield: 75.00%; mp: 142.6–143.6 °C; IR (KBr): υ 3215, 2924, 2842, 1646, 1569, 1509, 1475, 1393, 814 cm^−1^; ^1^H-NMR (400 MHz, Chloroform-*d*) δ 7.41 (dd, *J* = 1.8, 0.8 Hz, 1H), 7.35–7.27 (m, 2H), 6.88–6.80 (m, 2H), 6.41–6.31 (m, 2H), 6.26 (s, 1H), 4.62 (d, *J* = 5.2 Hz, 2H), 4.07 (s, 2H), 3.80 (s, 3H), 2.72 (s, 3H); ^13^C-NMR (101 MHz, Chloroform-*d*) δ 162.95, 159.74, 159.17, 158.65, 155.44, 150.25, 142.42, 139.16, 130.54, 126.98, 113.90, 113.14, 110.53, 108.18, 55.23, 37.05, 36.33, 12.33; HRMS (*m*/*z*): calcd. for C_20_H_19_N_4_O_4_S (M + H^+^) 411.11270, found 411.11199.

*N-(2-chlorobenzyl)-6-(4-methoxybenzyl)-3-methyl-7-oxo-7H-thiazolo[3,2-b]-1,2,4-triazine-2-carboxamide* (**5j**): White solid; Yield: 71.50%; mp: 156.4–172.2 °C; IR (KBr): υ 3258, 1656, 1610, 1570, 1512, 1350, 1300, 1036, 752 cm^−1^; ^1^H-NMR (400 MHz, Chloroform-*d*) δ 7.49–7.38 (m, 2H), 7.34–7.24 (m, 4H), 6.88–6.80 (m, 2H), 6.52 (s, 1H), 4.70 (d, *J* = 5.3 Hz, 2H), 4.06 (s, 2H), 3.79 (s, 3H), 2.71 (s, 3H); ^13^C-NMR (101 MHz, Chloroform-*d*) δ 162.92, 159.71, 158.99, 158.67, 155.58, 138.96, 134.44, 133.70, 130.67, 130.58, 129.73, 129.53, 127.30, 127.01, 113.93, 112.98, 55.25, 42.46, 36.34, 12.30; HRMS (*m*/*z*): calcd. for C_22_H_20_ClN_4_O_3_S (M + H^+^) 455.09446, found 455.07662.

### 3.3. Crystal Structure Determination

The crystals suitable for X-ray single-crystal structure study were grown by slow evaporation from methanol solution for compound **3e**. Data collections were performed on the Bruker AXS Smart APEX II CCD X–diffractometer equipped with graphite monochromated Mo Kα radiation (*λ* = 0.71073 Å) at 296 ± 2K. The crystal structure was determined by direct methods and refined by full–matrix least squares fitting on *F*^2^ by SHELXS–97 [30]. All non-hydrogen atoms were refined anisotropically.

The crystallographic data have been deposited at the Cambridge Crystallographic Data Centre. These data can be obtained free of charge at http://www.ccdc.cam.ac.uk/conts/retrieving.html (or from the CCDC, 12 Union Road, Cambridge CB2 1EZ, UK; Fax: +44 1223 336033; E-mail: deposit@ccdc.cam.ac.uk).

### 3.4. Determination of MIC for Bacterial Strains

The newly prepared compounds were screened for their antibacterial and antitubercular activity against *S. aureus*, *B. subtilis*, *E. coli*, *P. aeruginosa*, and *M. smegmatis*. The strains used in antibacterial tests were obtained from the National Center for Medical Culture Collection, China. A standard inoculum (1.5 × 10^7^ c.f.u/ml 0.5 McFarland standards) was introduced onto the surface of sterile agar plates and spread with a sterile glass spreader. The samples were dissolved in methanol at a concentration of 2 mg/mL. The MIC value was determined using a series dilution method. The diluted solutions in Mueller Hinton Broth were dispensed into the wells of a microtiter plate, and then an aliquot of 5 × 10^5^ c.f.u/mL of bacterial suspension was added to each well. The value of MIC was determined after 24 h of incubation at 37 °C.

### 3.5. Pharmacological Target Profiling

Pharmacophore screening was carried out using the Ligand Profiler protocol in Discovery Studio 3.5 software. The diverse conformations of the active compound were generated by using the Prepare Ligand protocol. The generated conformations were regarded as a query to map with the pharmacophoric features of the selected database by a flexible 3D searching method. The pharmacophore model contained different antibacterial and antitubercular targets. Other operations were performed using default parameters.

### 3.6. Aminoacylation Assay

The LeuRS gene from *M. smegmatis* was cloned and expressed using vector pET28a(+) in *Escherichia coli* BL21 (DE3). The amount of *M. smegmatis* LeuRS required to catalyze the production of AMP from 1 μmol of ATP in 1 min at 37 °C was defined as one activity unit (1u). The expression product was purified according to the literature [31].

The aminoacylation assay was performed according to the literature [32,33]. All test compounds were dissolved in CH_3_OH. The reaction mixture normally consisted of 50 mM HEPES-KOH (pH 8.0), 30 mM MgCl_2_, 1 mM DTT, 30 mM KCl, 13 μM L-[^14^C]leucine (306 mCi/mmol), 15 μM *E. coli* tRNA, 0.2 pM *M. smegmatis* LeuRS, and 4 mM ATP, 0.02% BSA (wt/vol). The appropriate concentrations of test compounds were added to the reaction mixture, and then the aminoacylation reactions were incubated at 37 °C for 7 min after the addition of 4 mM ATP, and aliquots were quenched by the addition of 10% TCA (wt/vol). The inhibitory activity of the aminoacylation of *M. smegmatis* LeuRS was obtained by liquid scintillation counting.

## 4. Conclusions

In summary, a series of new 7-oxo-7*H*-thiazolo[3,2-*b*]-1,2,4-triazine-2-carboxylic acid derivatives was synthesized starting from the 6-substituted arylmethyl-3-mercapto-1,2,4-triazin-5-ones and ethyl 2-chloroacetoacetate. The intermediate β-keto ethers **2a**–**2e** underwent intramolecular cyclization in PPA on heating to give 3-methyl-7-oxo-7*H*-thiazolo[3,2-*b*]-1,2,4-triazines **3a**–**3e**. The higher nucleophilicity at the *N*2 position of 1,2,4-triazine ring led to the regioselectivity of the cyclization step, and the selective annulation was confirmed by the single-crystal X-ray crystallographic structure of compound **3e**. Further hydrolysis of these esters gave the corresponding acids, which were then amidated with various substituted arylmethylamines to obtain the amide derivatives. According to the results of biological evaluation, carboxylic acid derivatives or their corresponding ethyl esters have less of an effect on antibacterial properties, whereas compounds containing the terminal amide fragment exhibit better antibacterial and antitubercular activity. Particularly, compound **5f** displayed a significant antitubercular effect with MIC values of 50 μg/mL against *M. smegmatis* and better inhibition efficiency of *M. smegmatis* LeuRS (percent inhibition of 78.24 ± 4.05% at 15 μg/mL). As a result, it was found that the novel 7-oxo-7*H*-thiazolo[3,2-*b*]-1,2,4-triazine-2-carboxylic acid derivatives containing the terminal amide fragment could be studied as LeuRS inhibitors for further biological research.

## Figures and Tables

**Table 1:** Optimization for the synthesis of compounds **3a**–**3e**.

Entry	Reaction Condition			
	Conventional Heating		MW-Irradiation	
	Time/min	Yield */%	Time/min	Yield */%
**3a**	60	68.86	20	81.24
**3b**	60	70.41	20	85.12
**3c**	60	71.31	20	83.78
**3d**	60	66.28	20	80.12
**3e**	60	75.26	20	87.66

* isolated yield.

**Table 2:** X-ray crystallographic data for compound **3e**.

CCDC number	1435646
Formula	(C_17_H_17_N_3_O_4_S)_4_·CH_3_OH
Formula weight	1468.62
Crystal color, shape	Colorless, block
Crystal system	Triclinic
Space group	*P*–1
*a*/Å	11.7136 (8)
*b*/Å	12.5137 (9)
*c*/Å	13.8247 (9)
*α* (°)	104.2510 (10)
β (°)	98.4010 (10)
*γ* (°)	110.7180 (10)
Volume/Å^3^	1775.2 (2)
Temperature/K	296 (2)
*Z*	1
Density (calculated)/g·cm^−3^	1.378
*F* (000)	769
Reflections collected/unique	10269/6238 [R(int) = 0.0197]
Goodness-of-fit of F^2^	1.137
Final *R* indices [*I* > 2*σ*(*I*)]	R1 = 0.0547, wR2 = 0.1655
*R* indices (all data)	R1 = 0.0821, wR2 = 0.1780
Largest diff. peak and hole/e.Å^−3^	0.448 and −0.401

**Table 3:** Antibacterial and antitubercular activity of the synthesized compounds. MIC: minimum inhibitory concentration.

Entry	Antibacterial Activity MIC (μg/mL)				Antitubercular Activity MIC (μg/mL)
	*S. aureus*	*B. subtilis*	*E. coli*	*P. aeruginosa*	*M. smegmatis*
**3a**	>800	>800	400	>800	>800
**3b**	100	>800	400	800	>800
**3c**	400	400	200	200	>800
**3d**	>800	800	400	400	>800
**3e**	>800	>800	>800	>800	>800
**4a**	200	>800	100	800	>800
**4b**	400	400	100	400	>800
**4c**	200	400	50	200	>800
**4d**	>800	>800	400	>800	>800
**4e**	>800	>800	800	>800	>800
**5a**	100	50	100	400	>800
**5b**	200	100	200	100	>800
**5c**	50	100	400	>800	>800
**5d**	50	200	50	100	>800
**5e**	100	50	400	400	>800
**5f**	200	200	100	200	50
**5g**	100	50	100	50	>800
**5h**	100	200	200	400	400
**5i**	200	400	200	100	>800
**5j**	200	200	400	400	>800
Ciprofloxacin	25	100	25	50	NT
Rifampicin	NT *	NT	NT	NT	25

* NT = Not tested.

**Table 4:** Identified potential antimicrobial targets of compound **5f**.

PDB Id	FitValue	Pharmacophore/Metadata/Target-Class C	Target
1TSL	0.774082	Methyltransferases	Bacterial thymidylate synthase
1OBH	0.347426	Aminoacyl-tRNA synthetases (AARSs)	Leucyl-tRNA synthetase
2HGZ	0.310616	Aminoacyl-tRNA synthetases (AARSs)	*p*-benzoyl-l-phenylalanyl-tRNA synthetase
1I2Z	0.0866648	Enoyl-[acyl-carrier-protein] reductase [NADH]	Bacterial enoyl acyl carrier protein reductase (FabI)
1LX6	0.0729051	Enoyl-[acyl-carrier-protein] reductase [NADH]	Bacterial enoyl-ACP reductase (FabI)
1MFP	0.058642	Enoyl-[acyl-carrier-protein] reductase [NADH]	Bacterial Enoyl-ACP reductases FabI and FabK
2B35	7.90584 × 10^−5^	Enoyl-[acyl-carrier-protein] reductase [NADH]	Mycobacterium tuberculosis enoyl reductase (InhA)

**Table 5:** Inhibitory effects of selected compounds on *M. smegmatis* LeuRS activity at 15 μg/mL.

Compound	Percent Inhibition (%)
**4b**	12.89 ± 2.31
**5f**	78.24 ± 4.05

## Supplementary Materials

^1^H-NMR and ^13^C-NMR spectra for the synthesized compounds are available online.

## Abbreviations

^1^H-NMR	proton nuclear magnetic resonance spectra
^13^C-NMR	carbon nuclear magnetic resonance spectra
AMP	adenosine monophosphate
ATP	adenosine triphosphate
CCDC	Cambridge Crystallographic Data Centre
DCM	dichloromethane
DMF	*N*,*N*-Dimethylformamide
DTT	dithiothreitol
EDCl	*N*′-(3-dimethylaminopropyl)-*N*-ethylcarbodiimide hydrochloride
HEPES	4-(2-hydroxyethyl)-1-piperazineethanesulfonic acid
HOBt	1-hydroxybenzotriazole
HRMS	high-resolution mass spectra
IC_50_	half maximal inhibitory concentration
IR	infrared spectra
KOH	potassium hydroxide
MIC	minimum inhibitory concentration
MS	mass spectra
ORTEP	Oak ridge thermal-ellipsoid plot program
PDB	protein data bank
PPA	polyphosphoric acid
SHELXS	the crystallographic programs for crystal structure solution and refinement written by G. M. Sheldrick
TCA	trichloroacetic acid
TLC	thin layer chromatography
TMS	tetramethylsilane
