# Supplementary material for: Synthesis, Characterization, and Biological Evaluation of Novel 7-Oxo-7H-thiazolo[3,2-b]-1,2,4-triazine-2-carboxylic Acid Derivatives
Source: Molecules. 2020 Mar 13;25(6):1307. doi: 10.3390/molecules25061307 (PMC7144117; doi:10.3390/molecules25061307)

Synthesis, characterization and biological evaluation of novel 7-oxo-7*H*-thiazolo[3,2-*b*]-1,2,4-triazine-2-carboxylic acid derivatives

Dong Cai [1], Tai Li [1], Qian Xie [1], Xiaofei Yu [1], Wei Xu [2], Yu Chen [2], Zhe Jin [1,*] and Chun Hu [1,*]

1 Key Laboratory of Structure-based Drug Design & Discovery, Ministry of Education , School of Pharmaceutical Engineering, Shenyang Pharmaceutical University, 110016, China;

2 School of Life Science and Biopharmaceutics, Shenyang Pharmaceutical University, Shenyang 110016, China;

* Correspondence: Zhe Jin (jinzheln@163.com) and Chun Hu (chunhu1999@163.com)

Spectroscopic data

^{1}H NMR of compounds **2a-3e** ………………..………………………….…S2-4
^{1}H NMR and ^{13}C NMR of compounds **3a-3e** ……..…………….………..…S5-9
^{1}H NMR and ^{13}C NMR of compounds **4a-4e** ……..…………..…………S10-14
^{1}H NMR and ^{13}C NMR of compounds **5a-5j** ……..………..……………..…S15-24

Ethyl 2-((6-(2-chlorobenzyl)-5-oxo-2,5-dihydro-1,2,4-triazin-3-yl)thio)-3-oxobutanoate (**2a**):

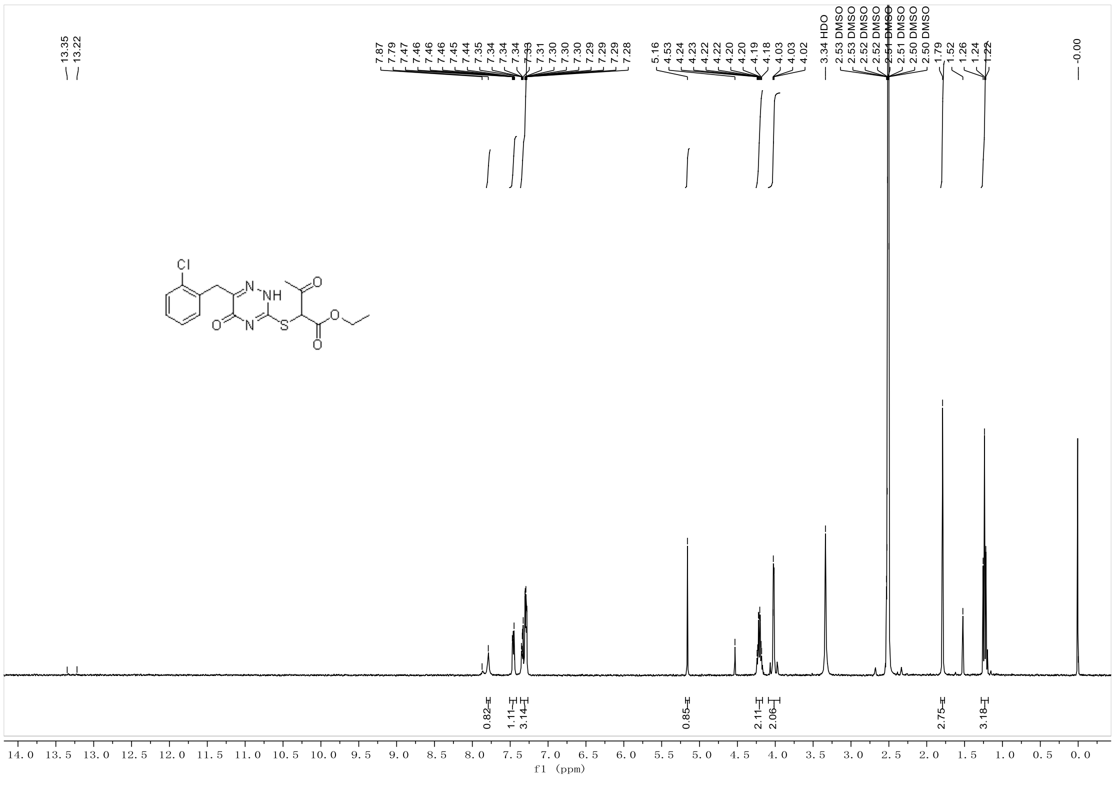

Ethyl 2-((6-(4-chlorobenzyl)-5-oxo-2,5-dihydro-1,2,4-triazin-3-yl)thio)-3-oxobutanoate (**2b**):

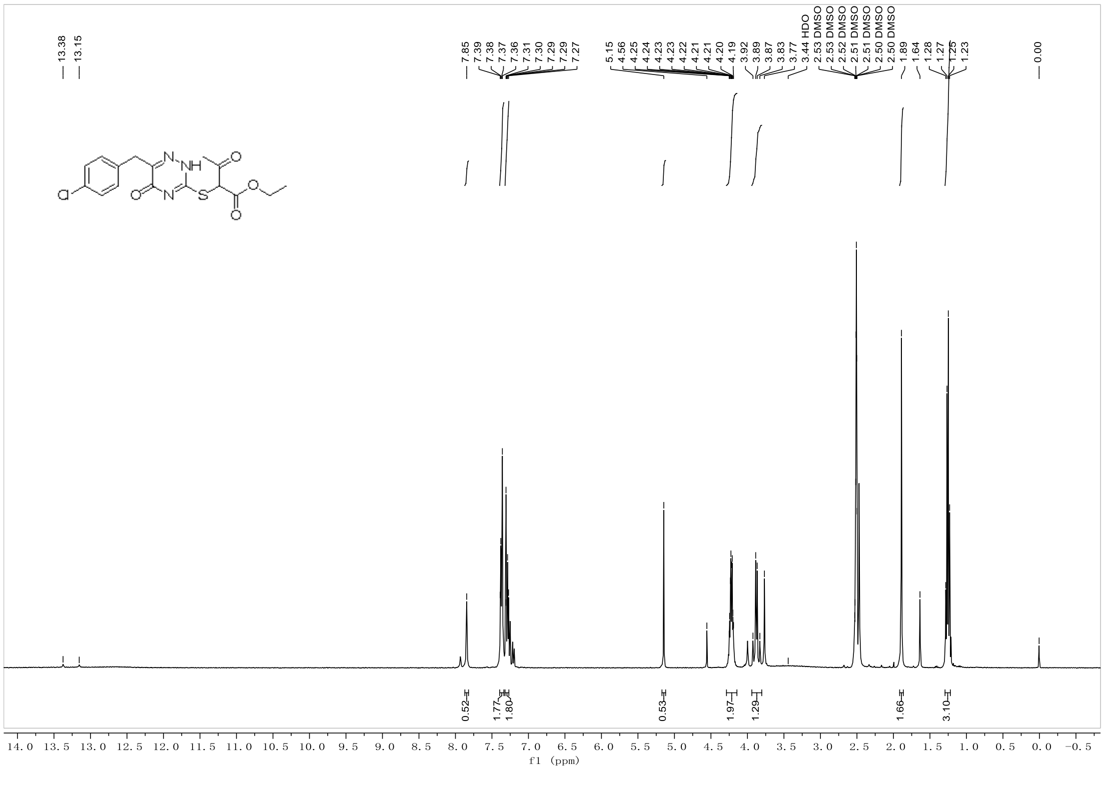

Ethyl 2-((6-(4-fluorobenzyl)-5-oxo-2,5-dihydro-1,2,4-triazin-3-yl)thio)-3-oxobutanoate (**2c**):

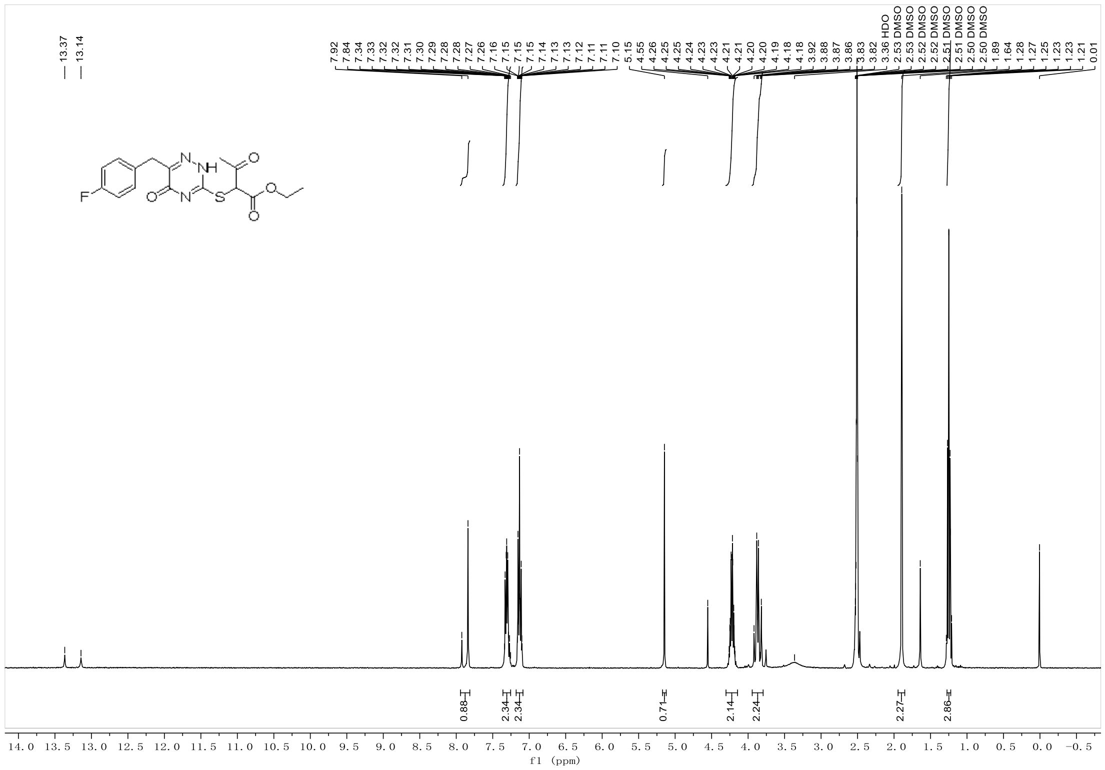

Ethyl 3-oxo-2-((5-oxo-6-(4-(trifluoromethyl)benzyl)-2,5-dihydro-1,2,4-triazin-3-yl)thio)butanoate (**2d**):

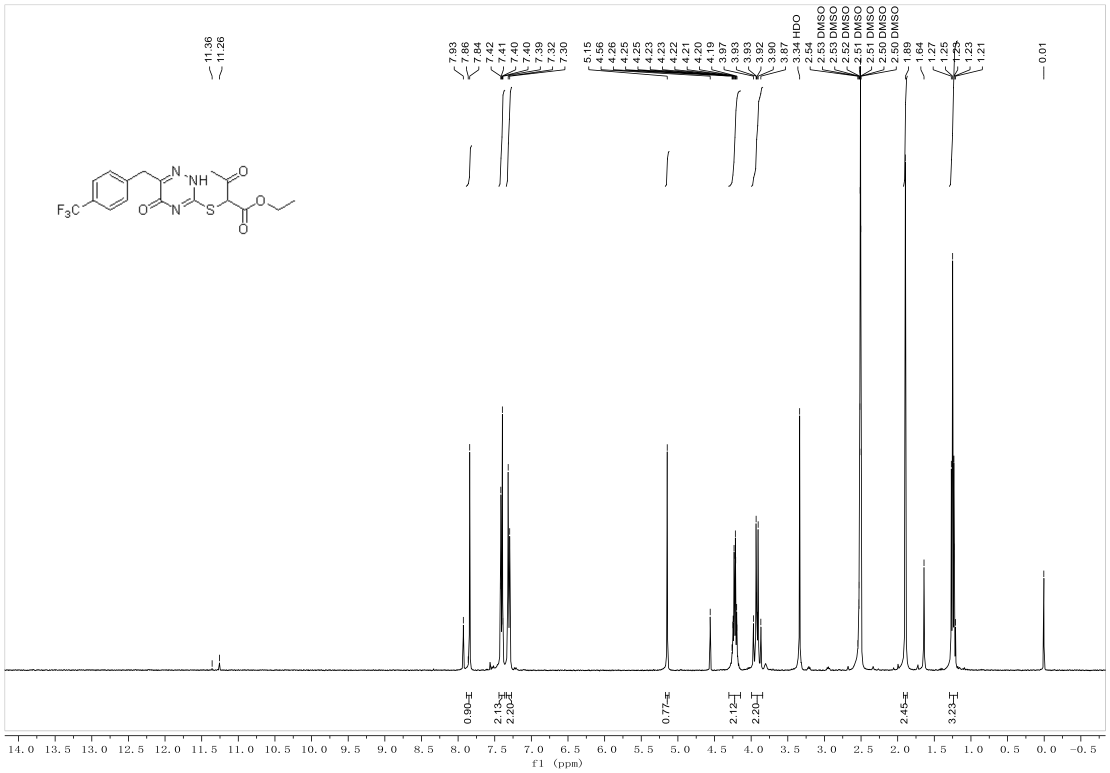

Ethyl 2-((6-(4-methoxybenzyl)-5-oxo-2,5-dihydro-1,2,4-triazin-3-yl)thio)-3-oxobutanoate (**2e**):

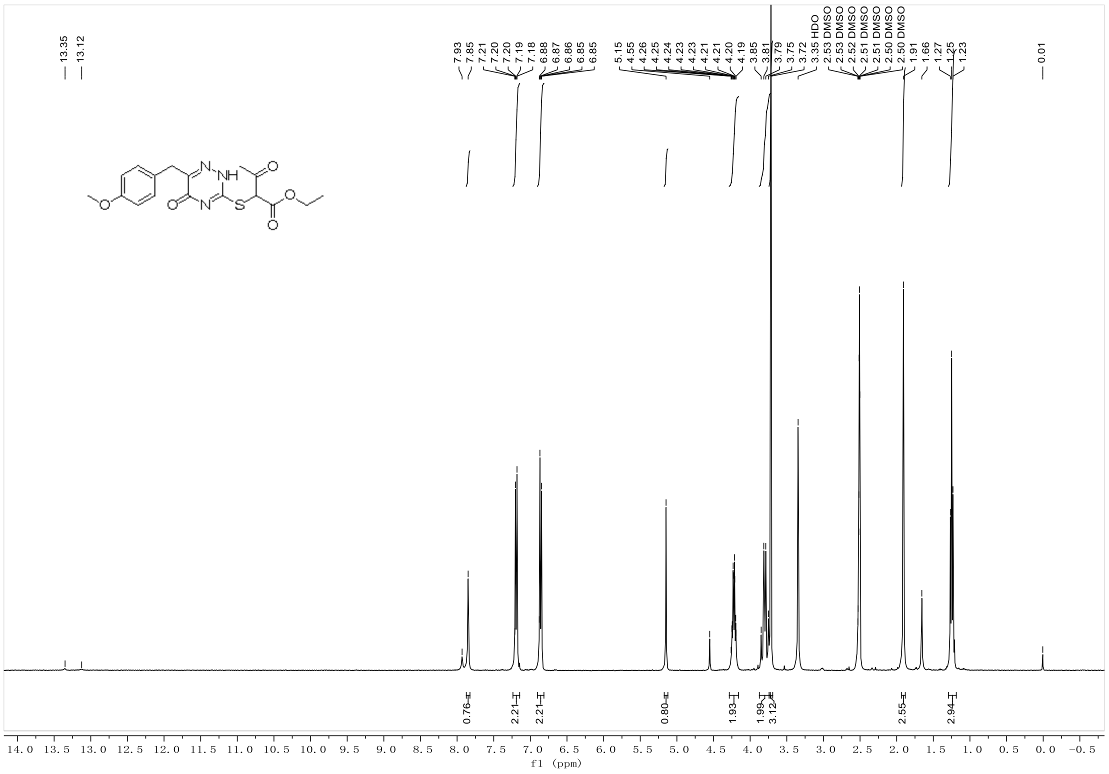

Ethyl 6-(2-chlorobenzyl)-3-methyl-7-oxo-7H-thiazolo[3,2-b]-1,2,4-triazine-2-carboxylate (**3a**):

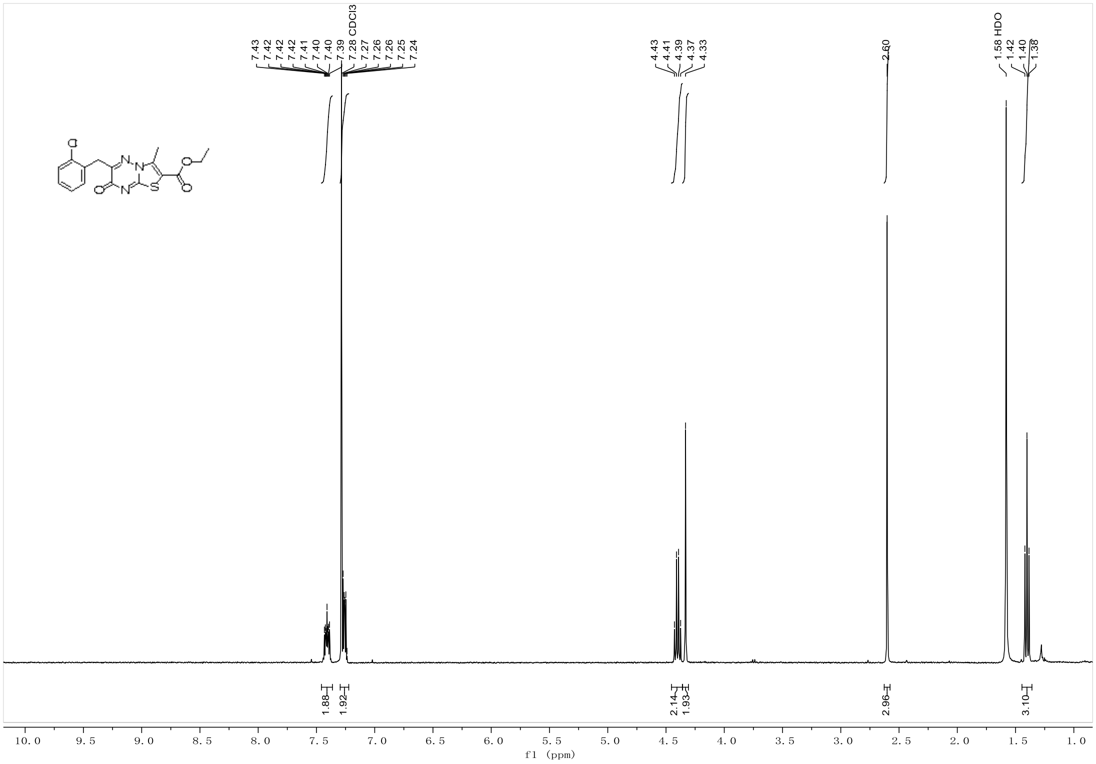

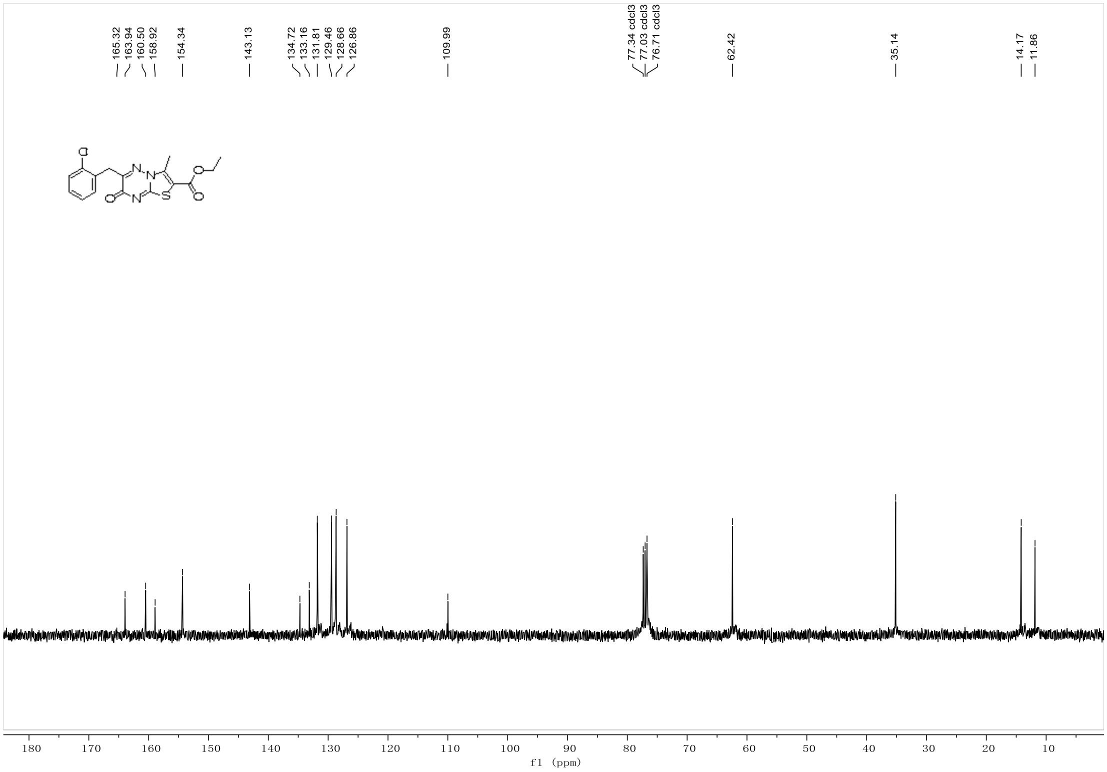

Ethyl 6-(4-chlorobenzyl)-3-methyl-7-oxo-7H-thiazolo[3,2-b]-1,2,4-triazine-2-carboxylate (**3b**):

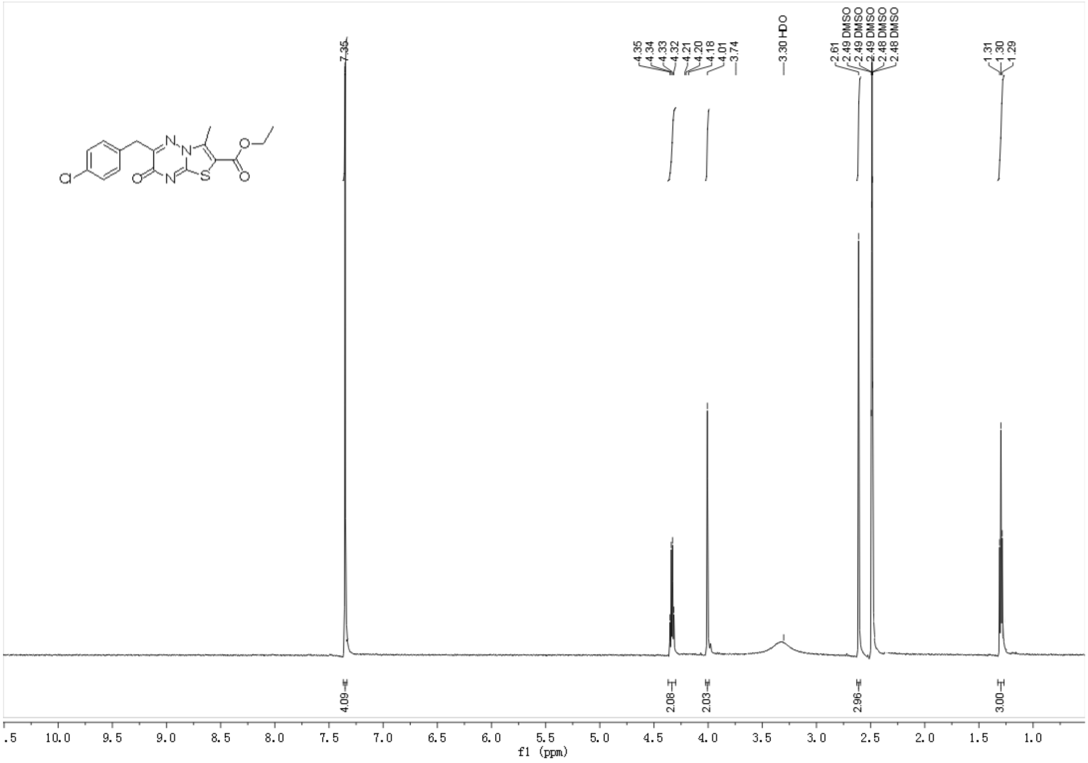

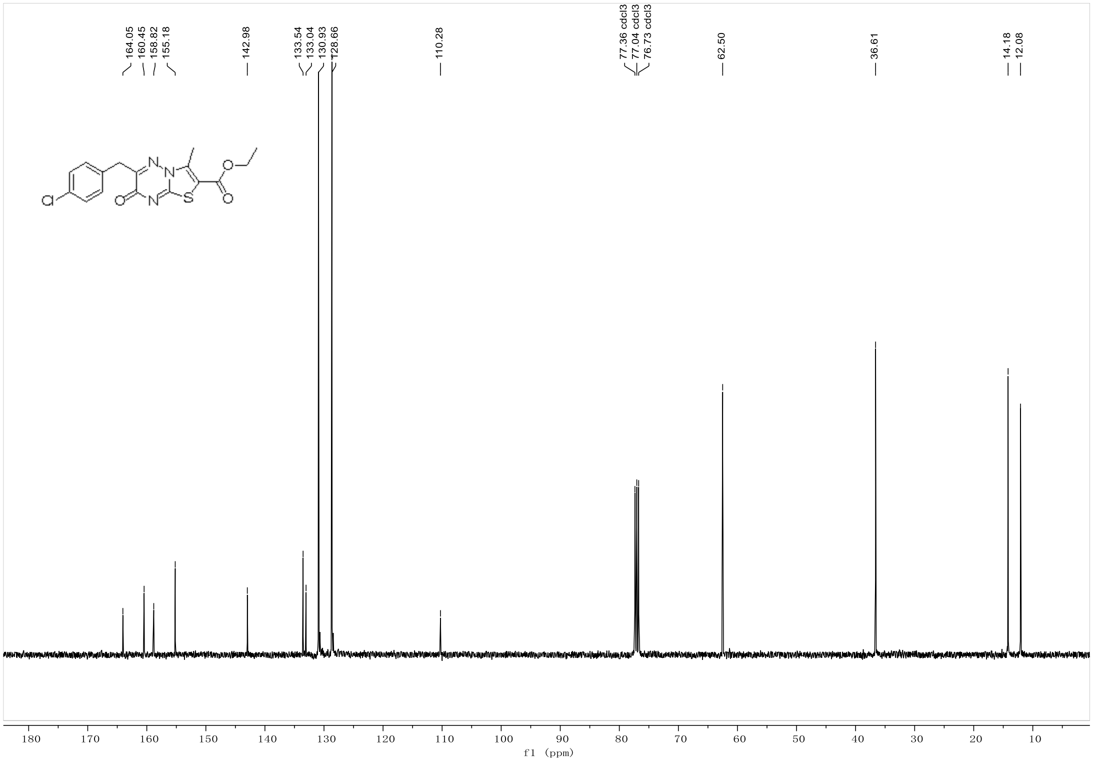

Ethyl 6-(4-fluorobenzyl)-3-methyl-7-oxo-7H-thiazolo[3,2-b]-1,2,4-triazine-2-carboxylate (**3c**):

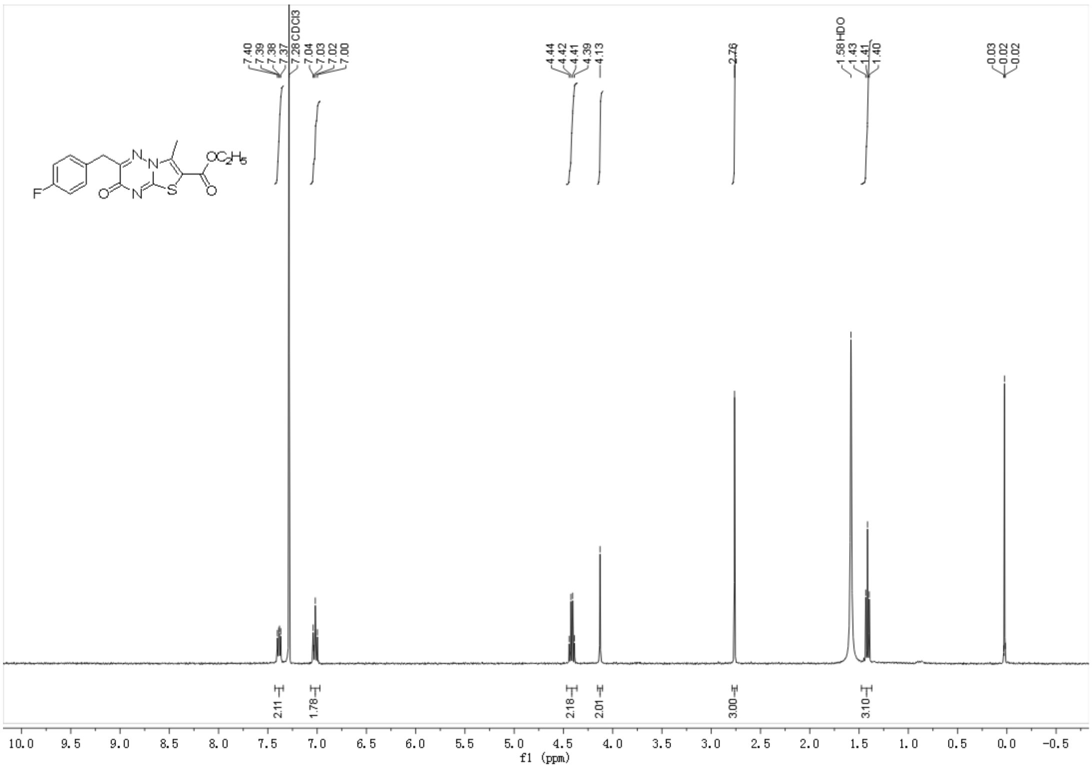

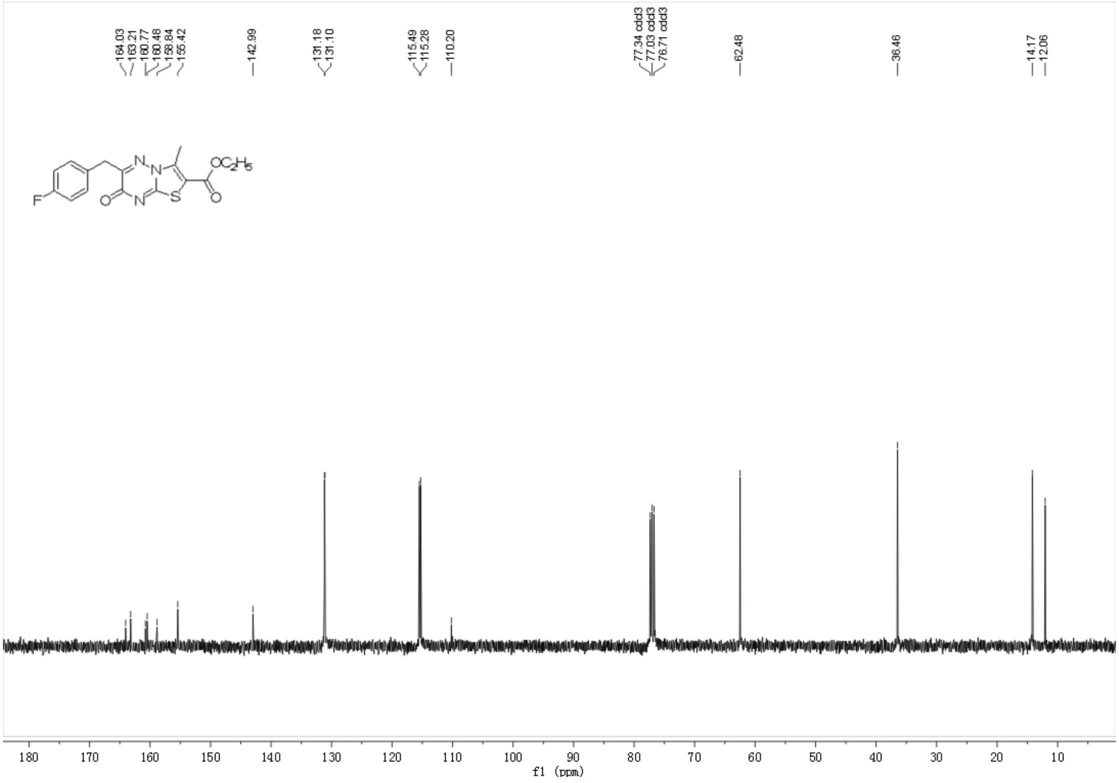

Ethyl 3-methyl-7-oxo-6-(4-(trifluoromethyl)benzyl)-7H-thiazolo[3,2-b]-1,2,4-triazine-2-carboxylate (**3d**):

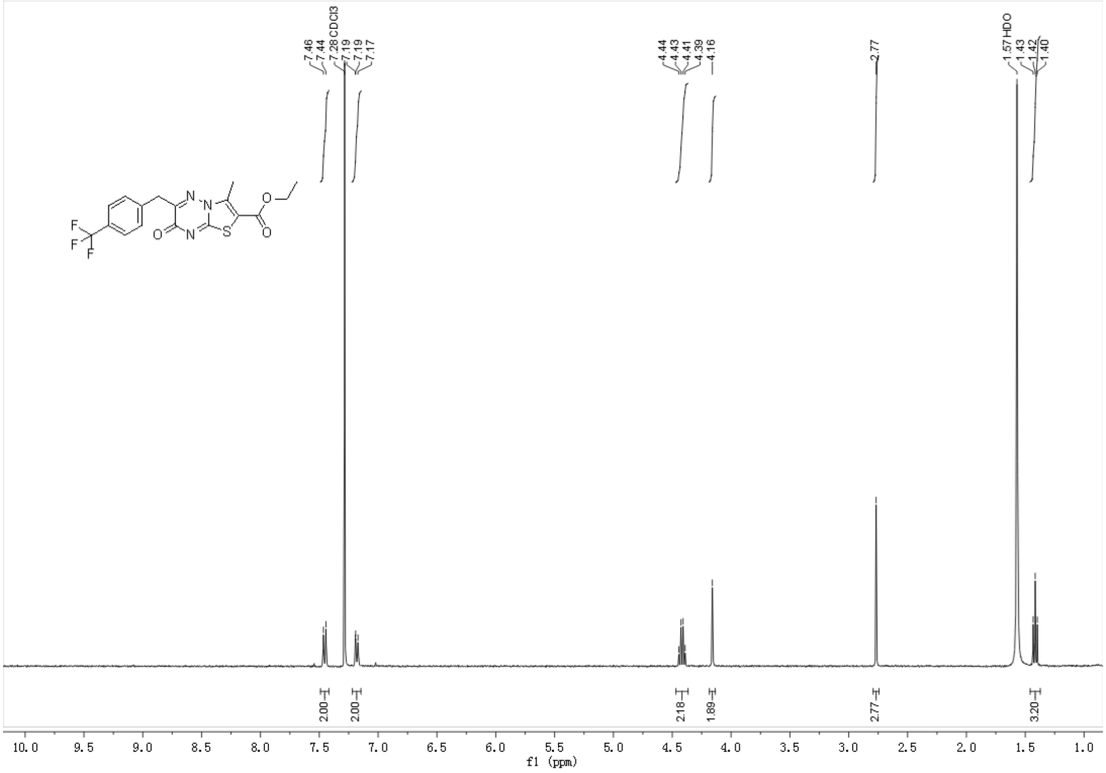

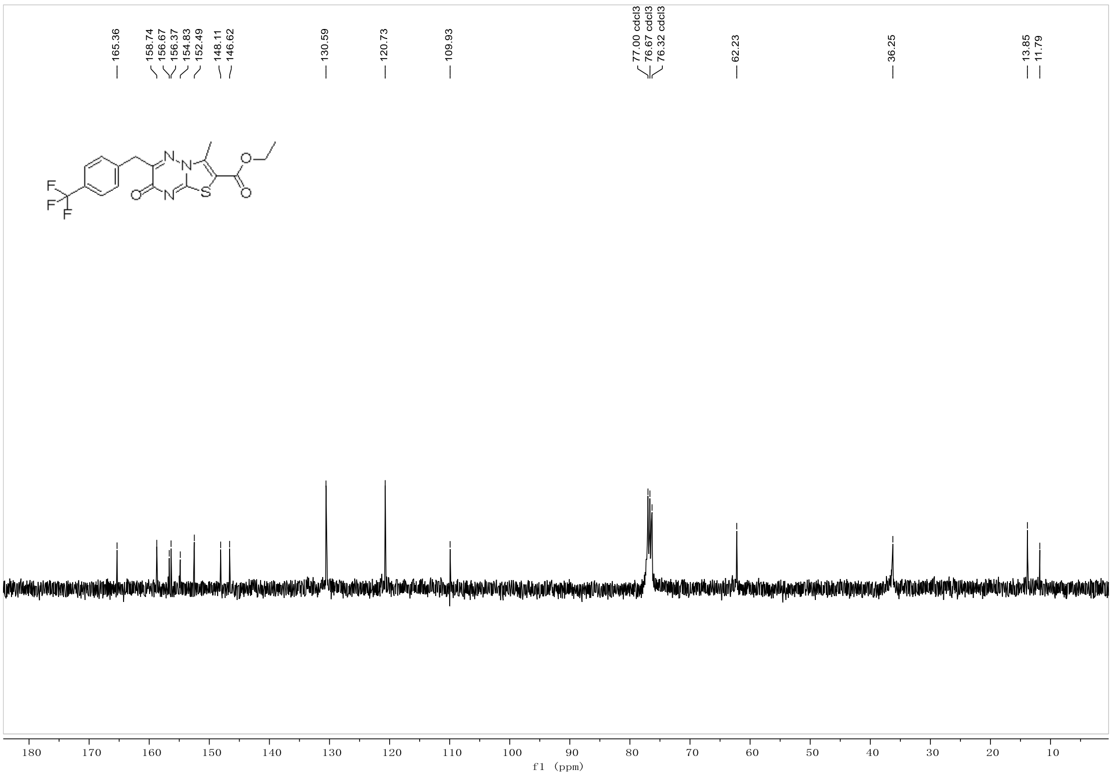

Ethyl 6-(4-methoxybenzyl)-3-methyl-7-oxo-7H-thiazolo[3,2-b]-1,2,4-triazine-2-carboxylate (**3e**):

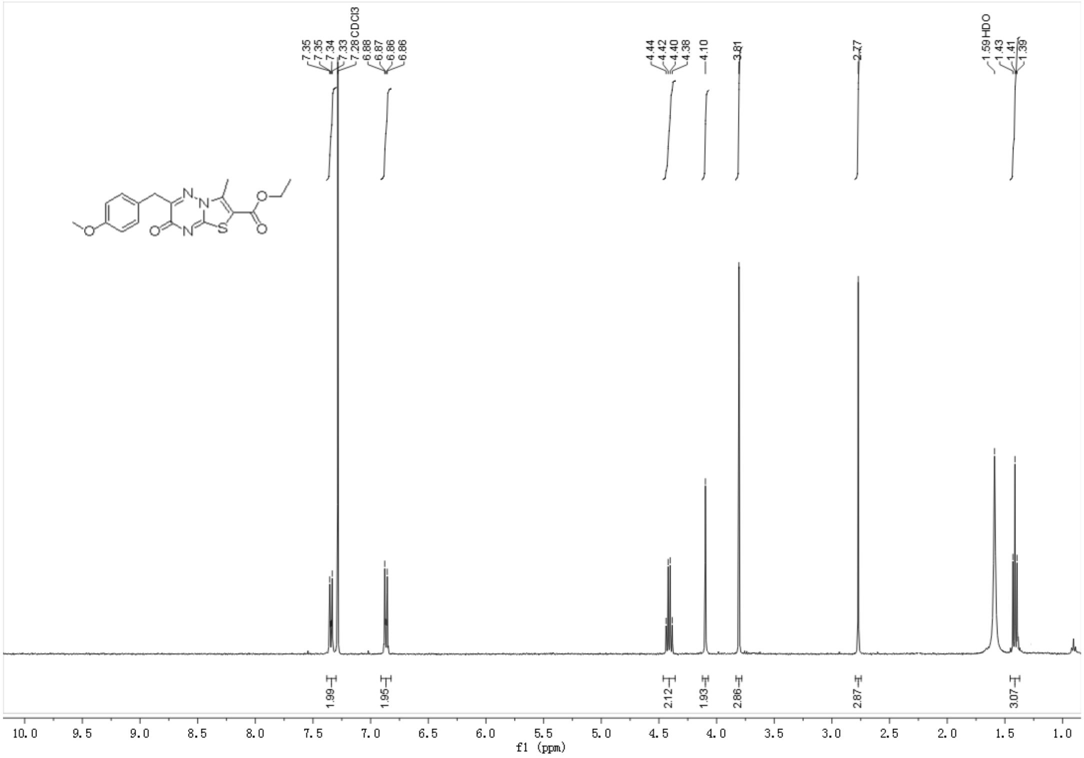

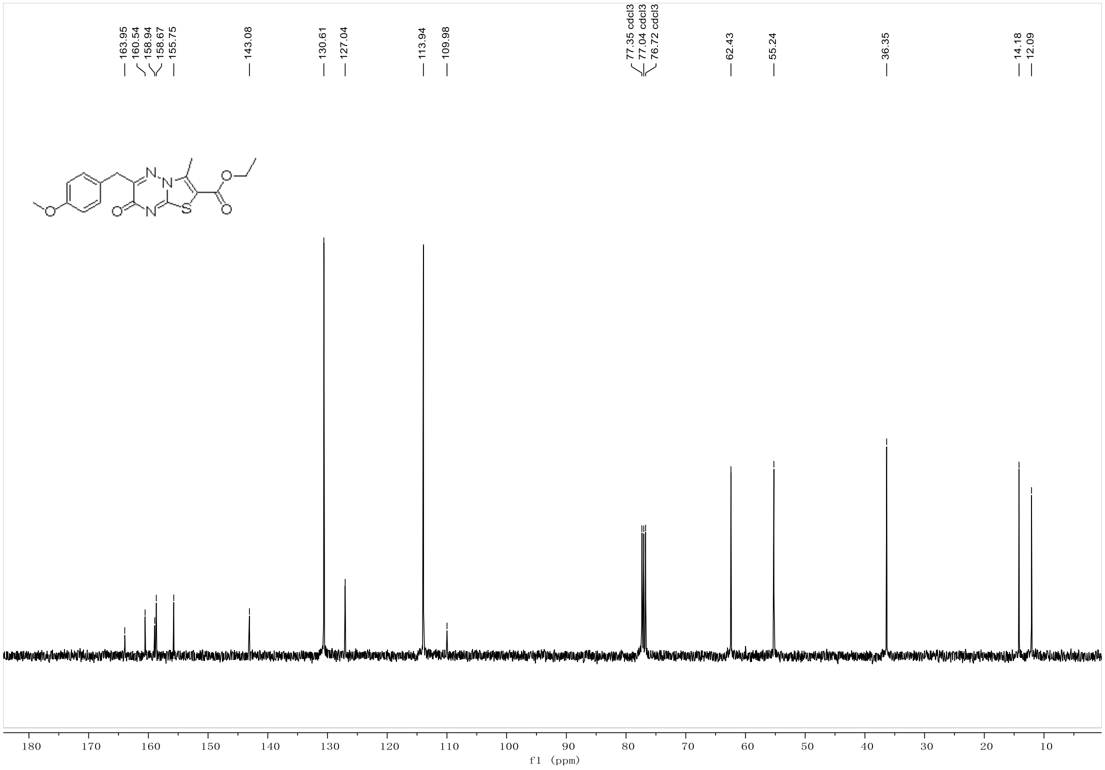

6-(2-Chlorobenzyl)-3-methyl-7-oxo-7H-thiazolo[3,2-b]-1,2,4-triazine-2-carboxylic acid (**4a**):

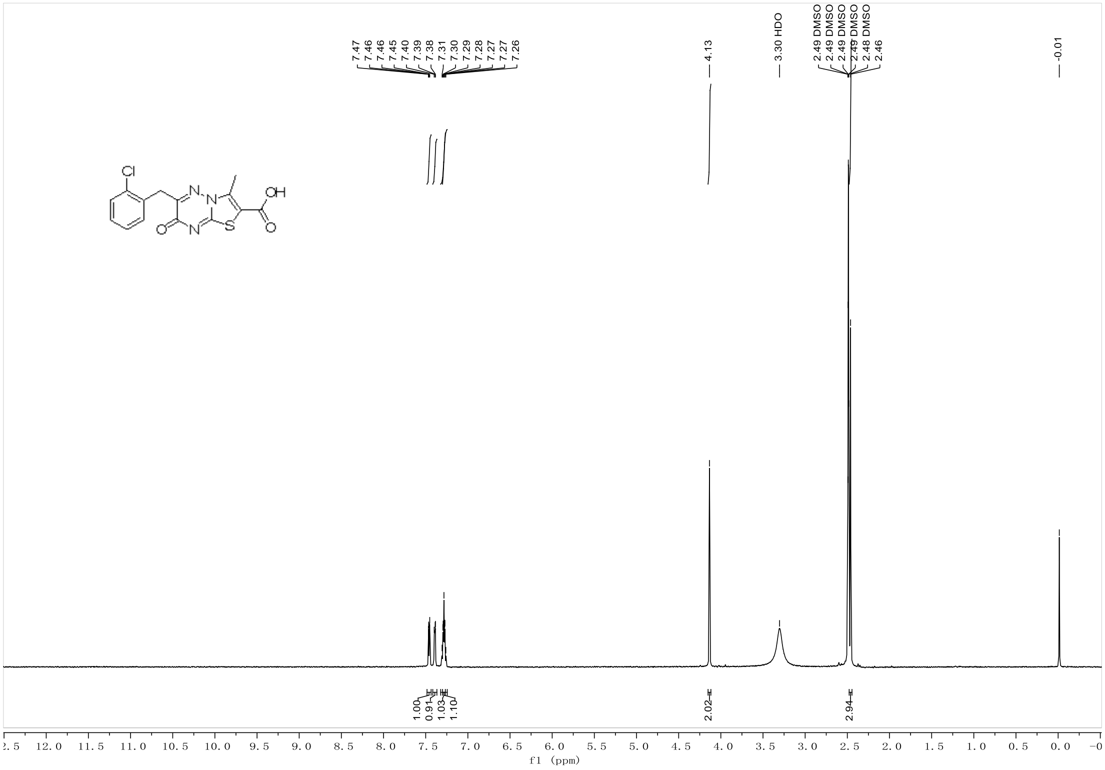

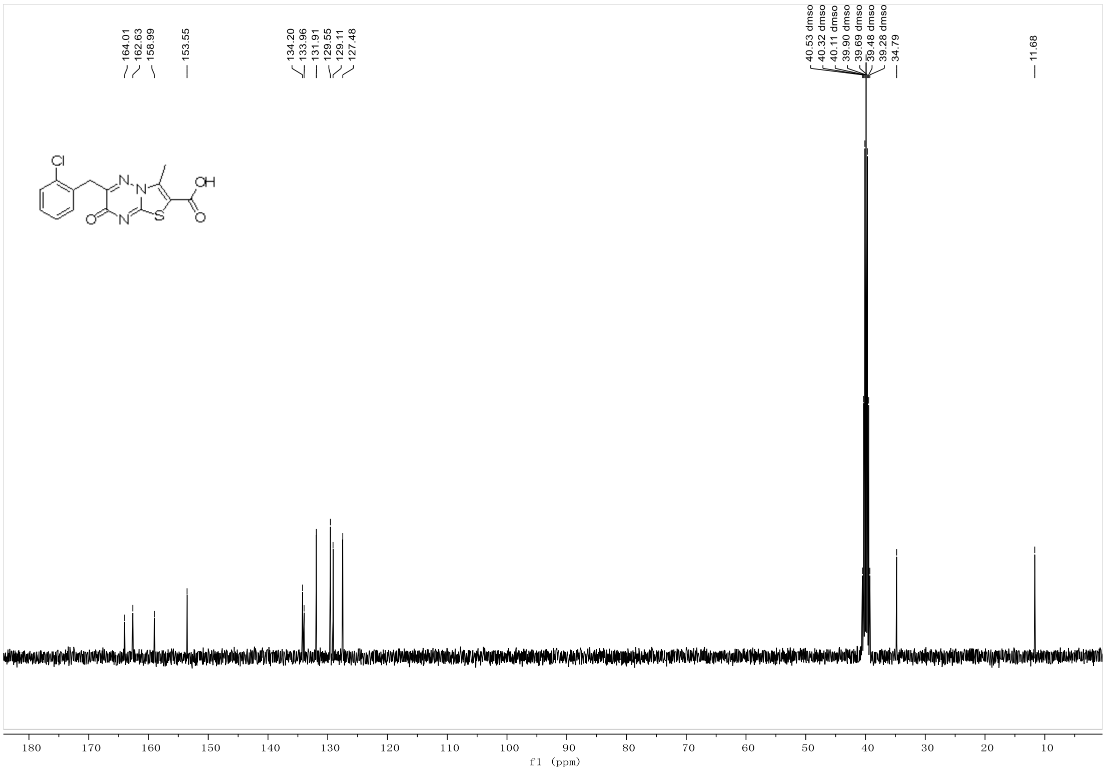

6-(4-Chlorobenzyl)-3-methyl-7-oxo-7H-thiazolo[3,2-b]-1,2,4-triazine-2-carboxylic acid (**4b**):

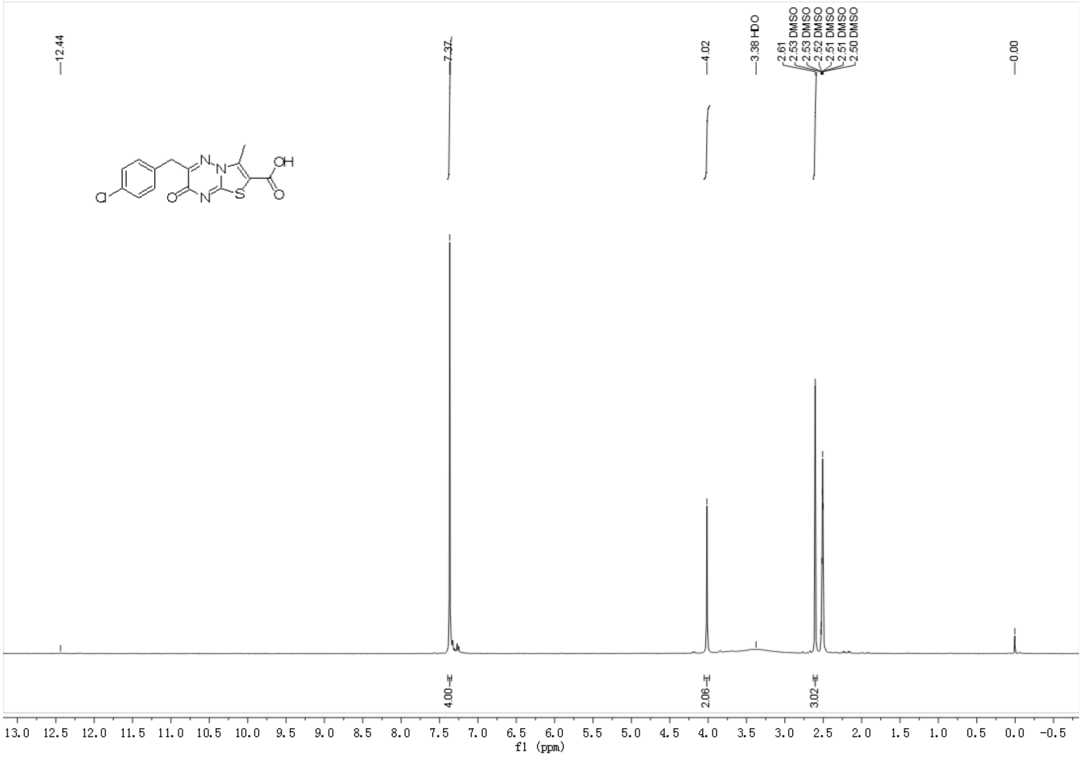

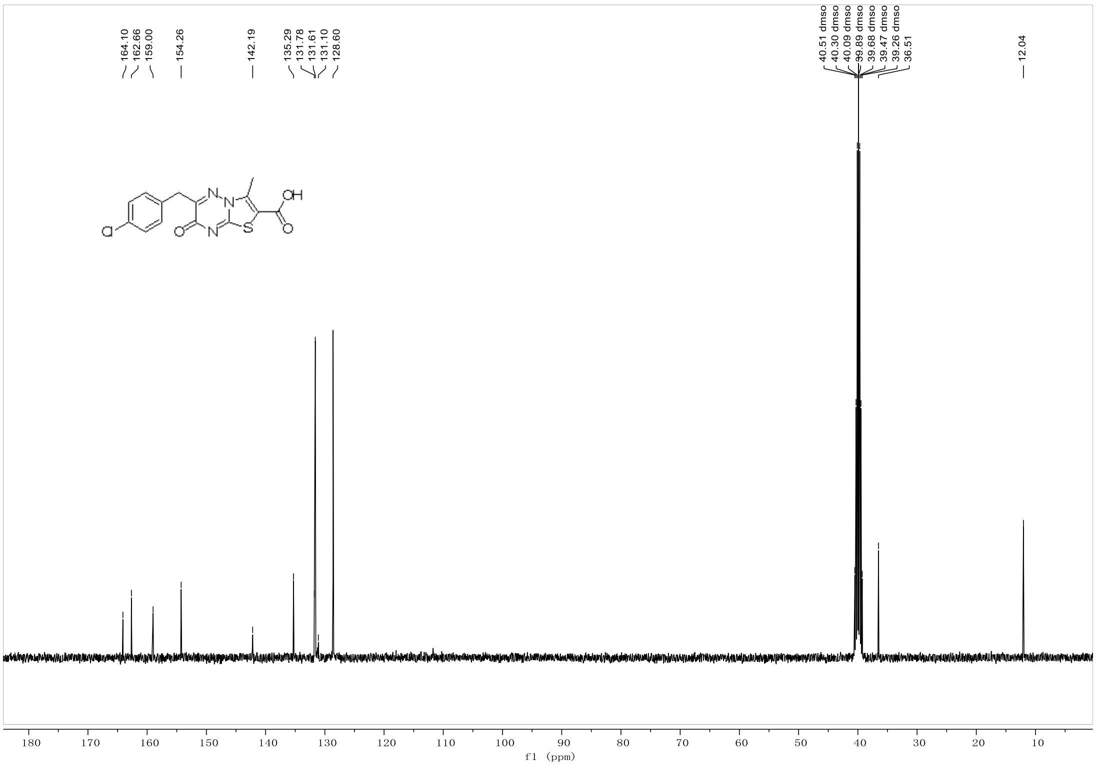

6-(4-Fluorobenzyl)-3-methyl-7-oxo-7H-thiazolo[3,2-b]-1,2,4-triazine-2-carboxylic acid (**4c**):

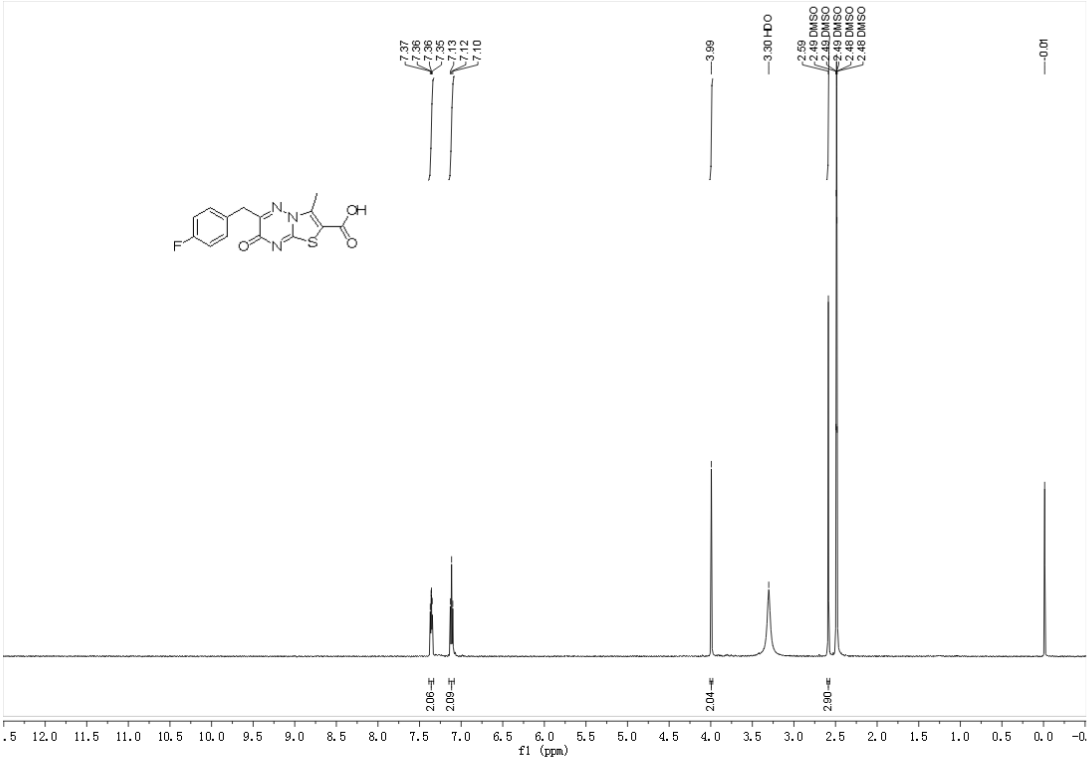

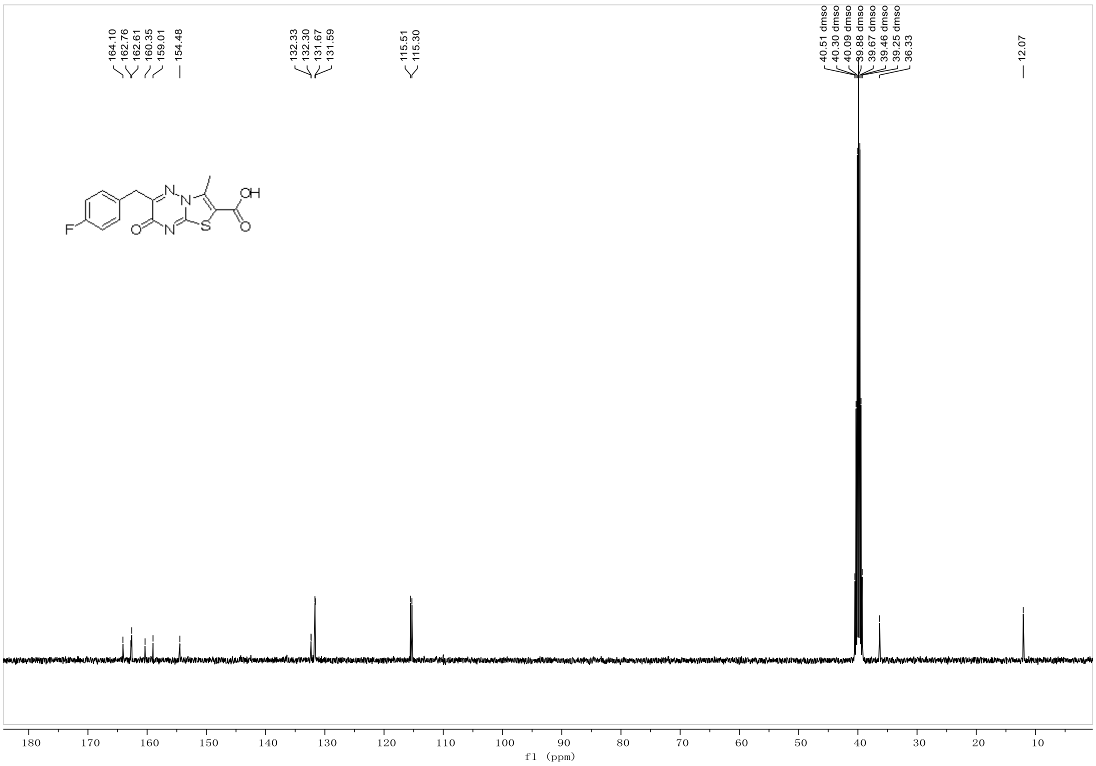

3-Methyl-7-oxo-6-(4-(trifluoromethyl)benzyl)-7H-thiazolo[3,2-b]-1,2,4-triazine-2-carboxylic acid (**4d**):

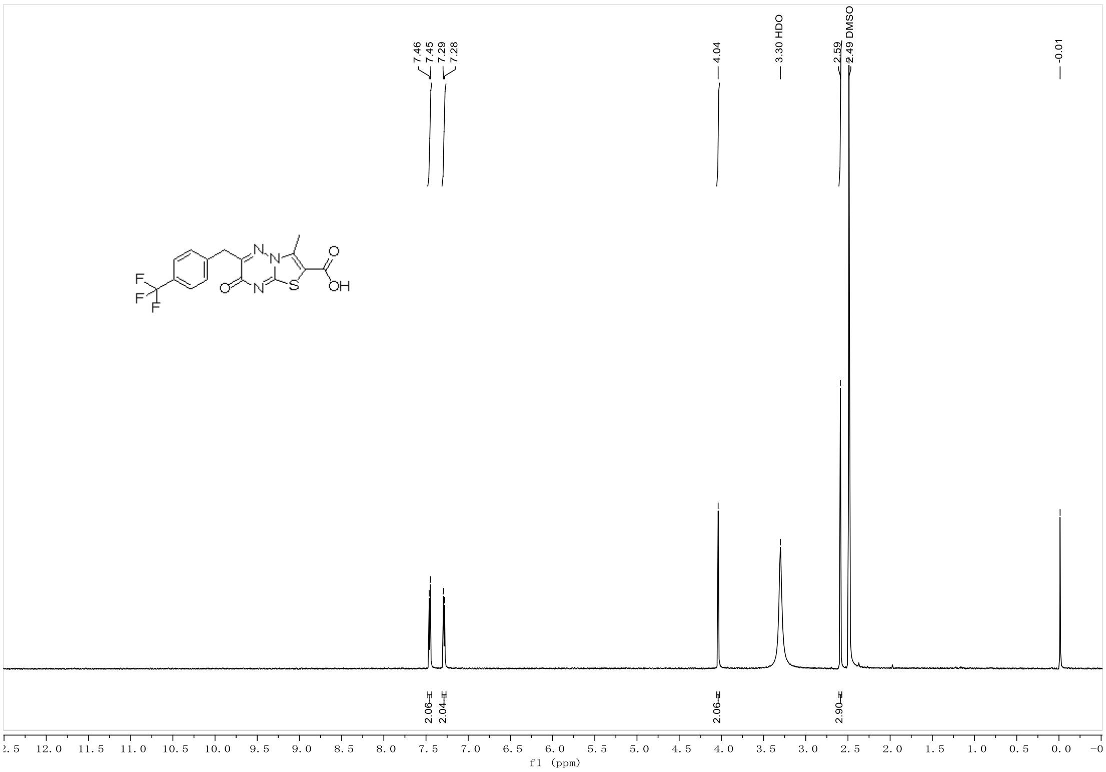

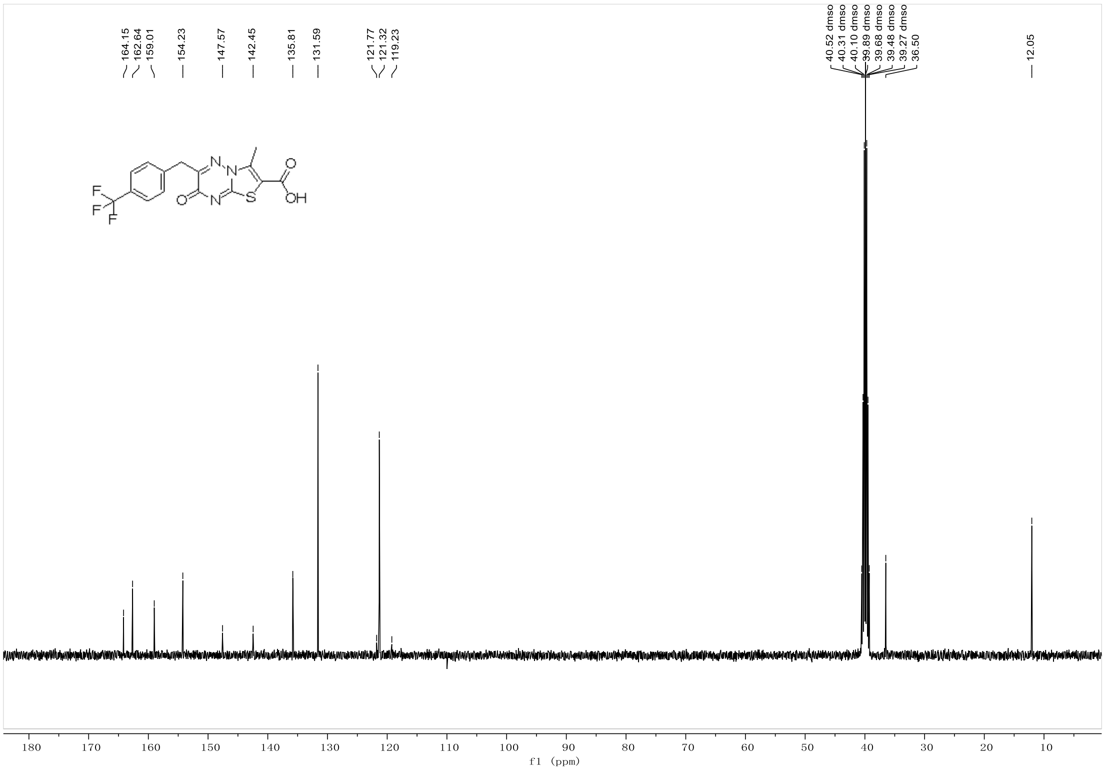

6-(4-Methoxybenzyl)-3-methyl-7-oxo-7H-thiazolo[3,2-b]-1,2,4-triazine-2-carboxylic acid (**4e**):

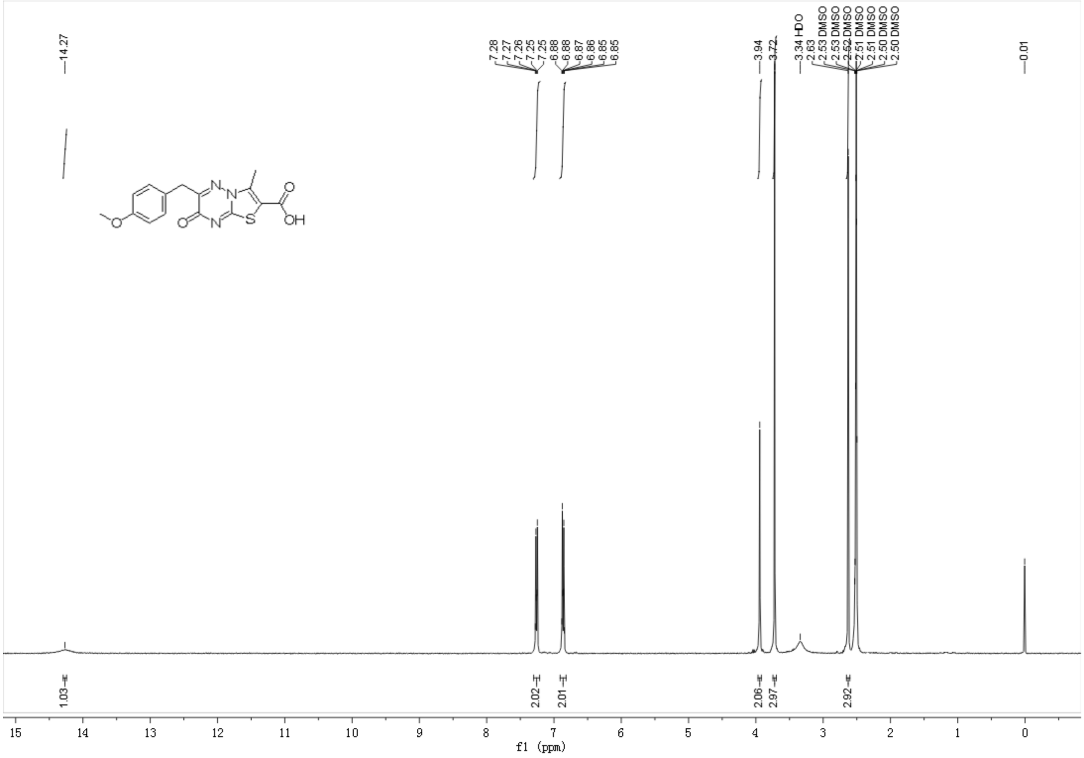

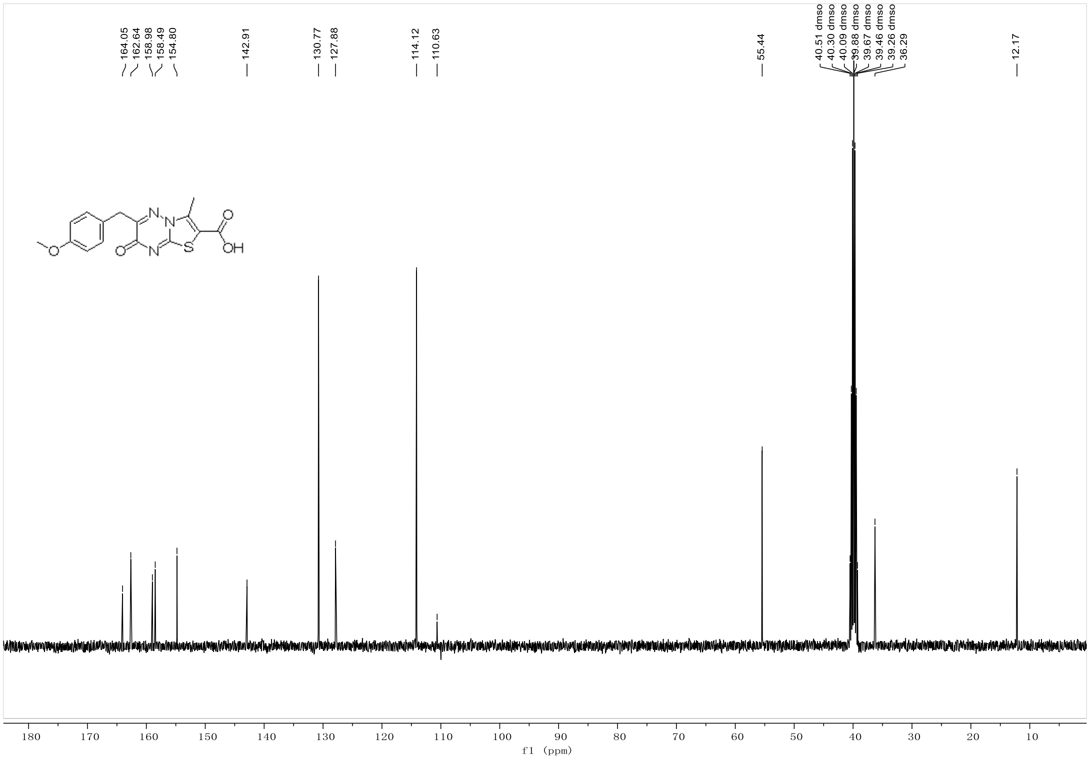

6-(2-Chlorobenzyl)-N-(furan-2-ylmethyl)-3-methyl-7-oxo-7H-thiazolo[3,2-b]-1,2,4-triazine-2-carboxamide (**5a**):

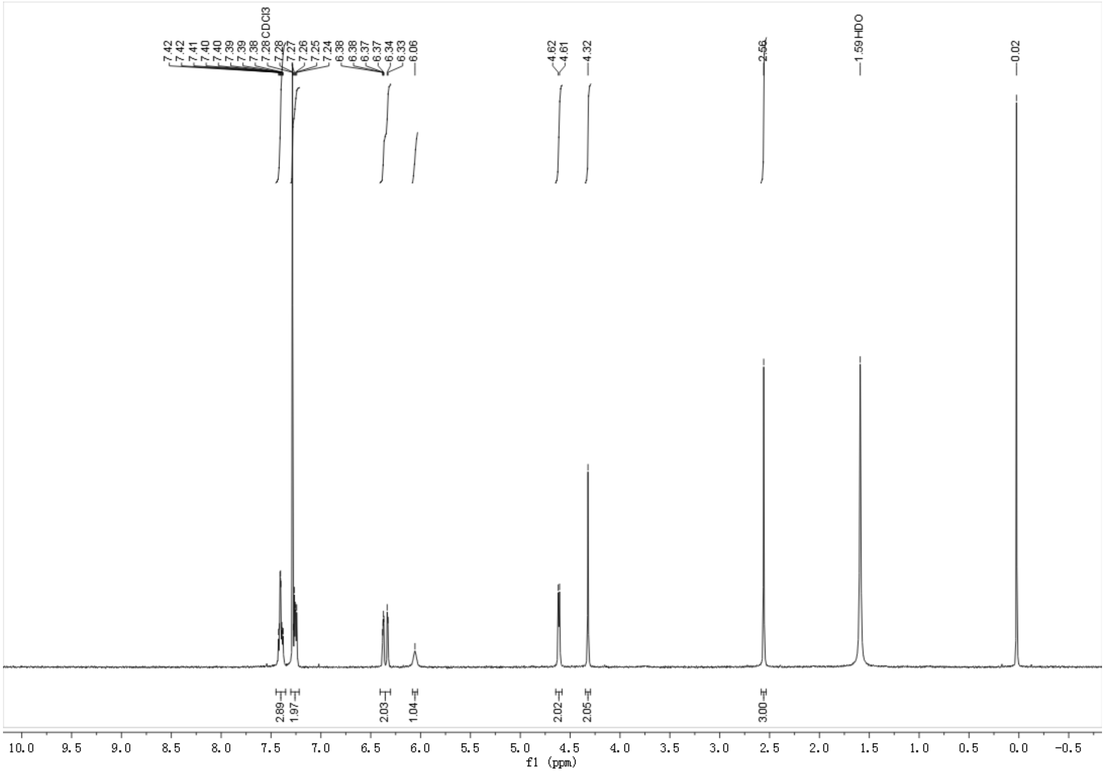

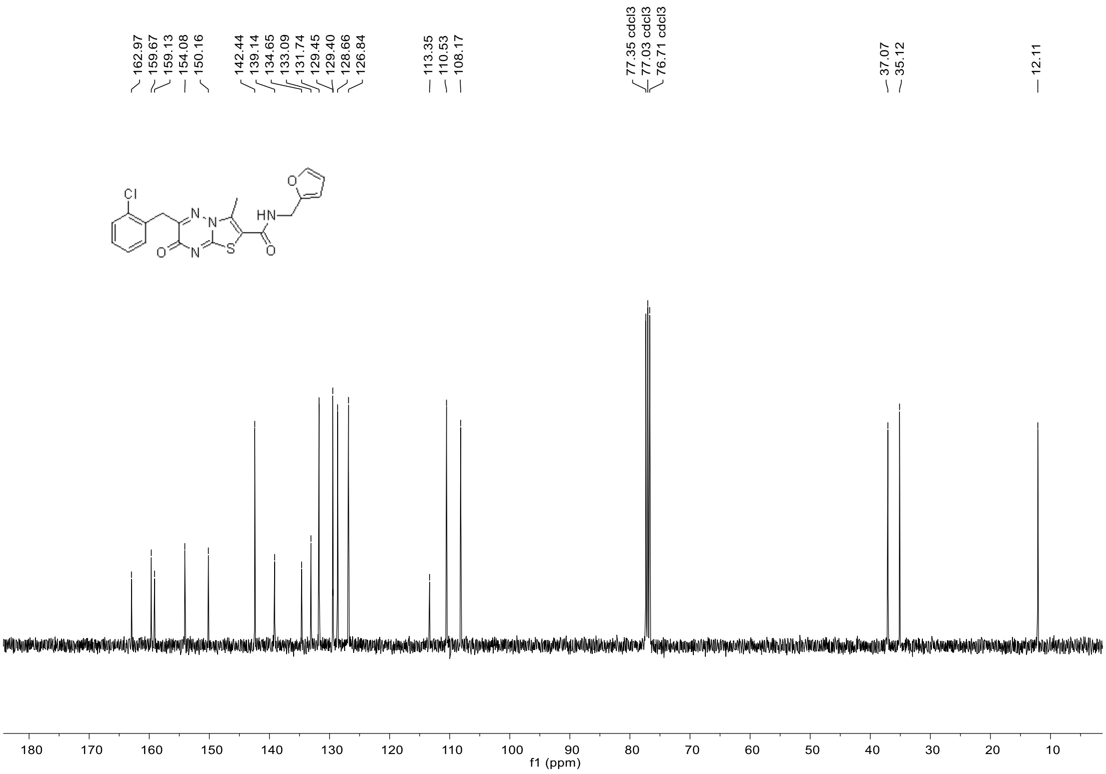

N,6-Bis(2-chlorobenzyl)-3-methyl-7-oxo-7H-thiazolo[3,2-b]-1,2,4-triazine-2-carboxamide (**5b**):

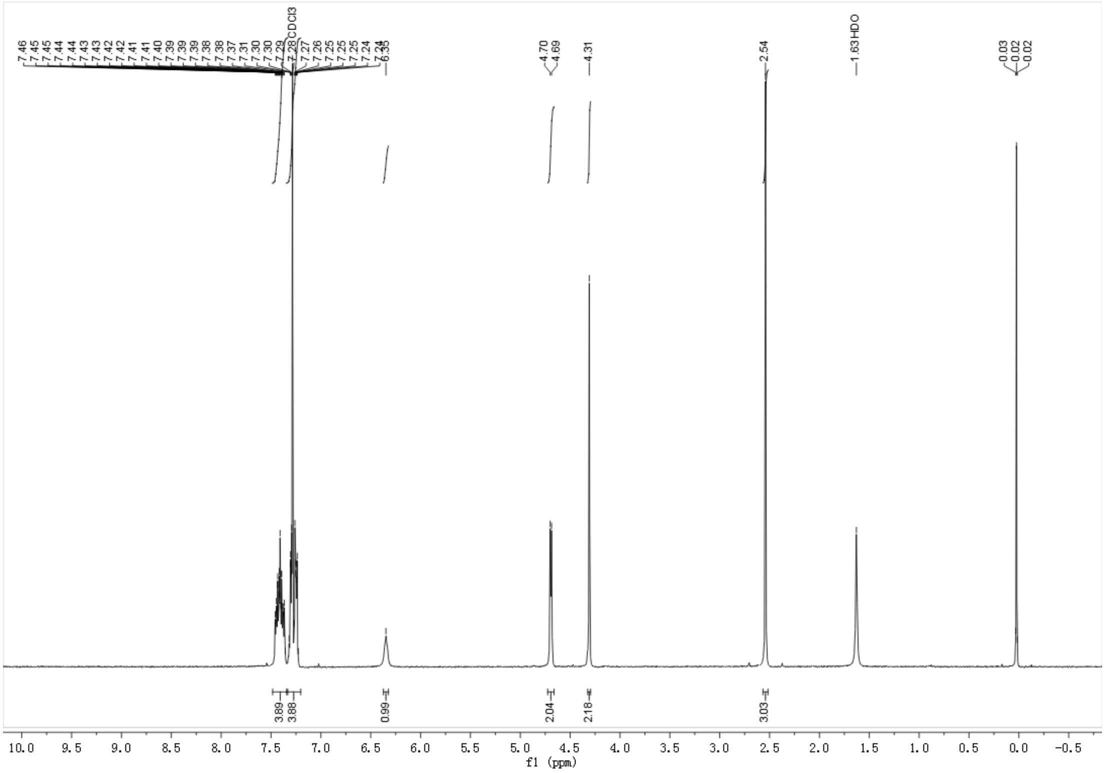

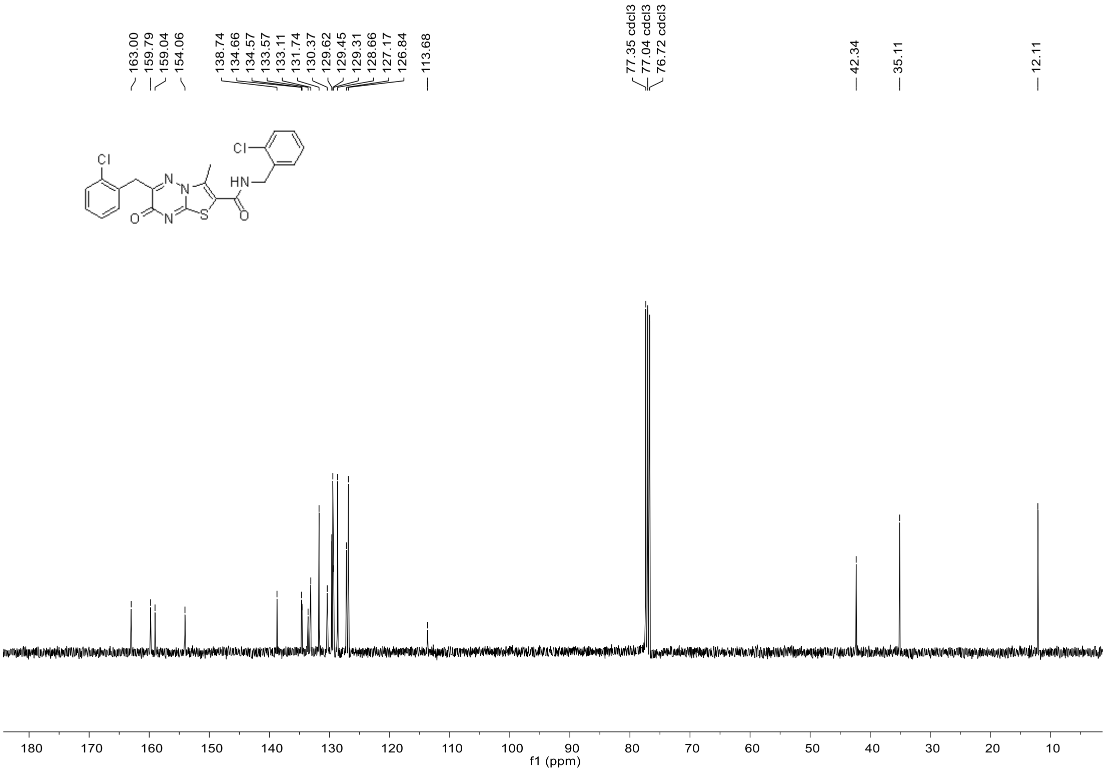

6-(2-Chlorobenzyl)-N-(2,4-dichlorobenzyl)-3-methyl-7-oxo-7H-thiazolo[3,2-b]-1,2,4-triazine-2-carboxamide (**5c**):

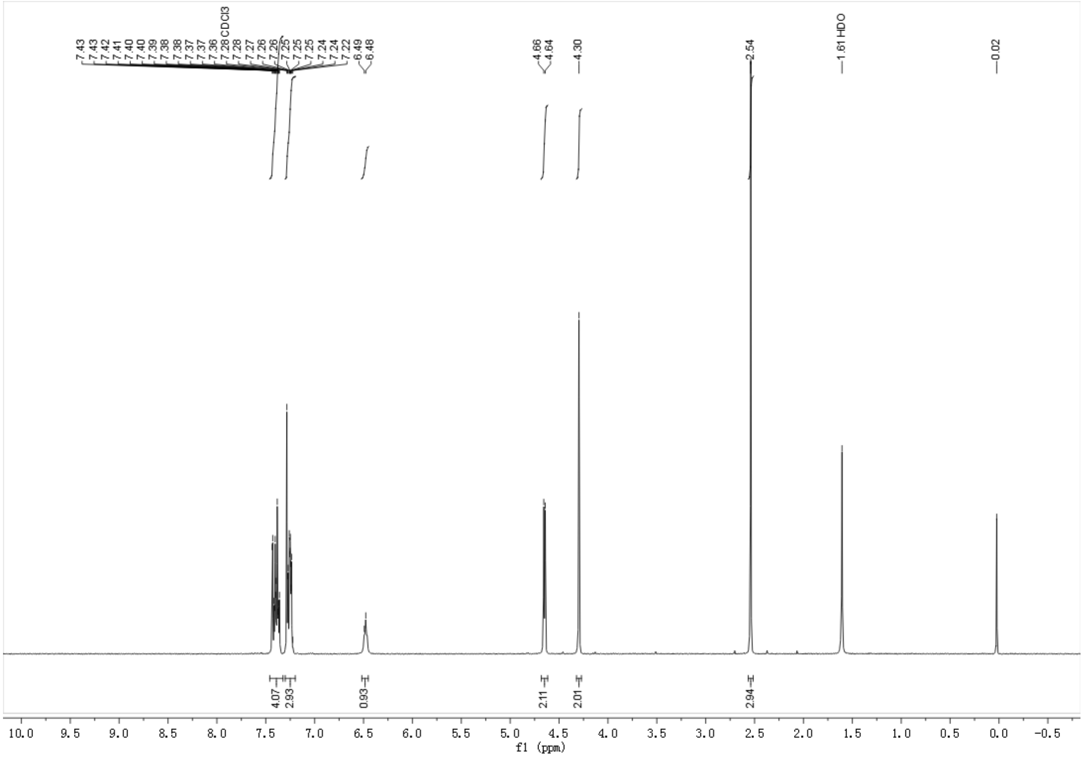

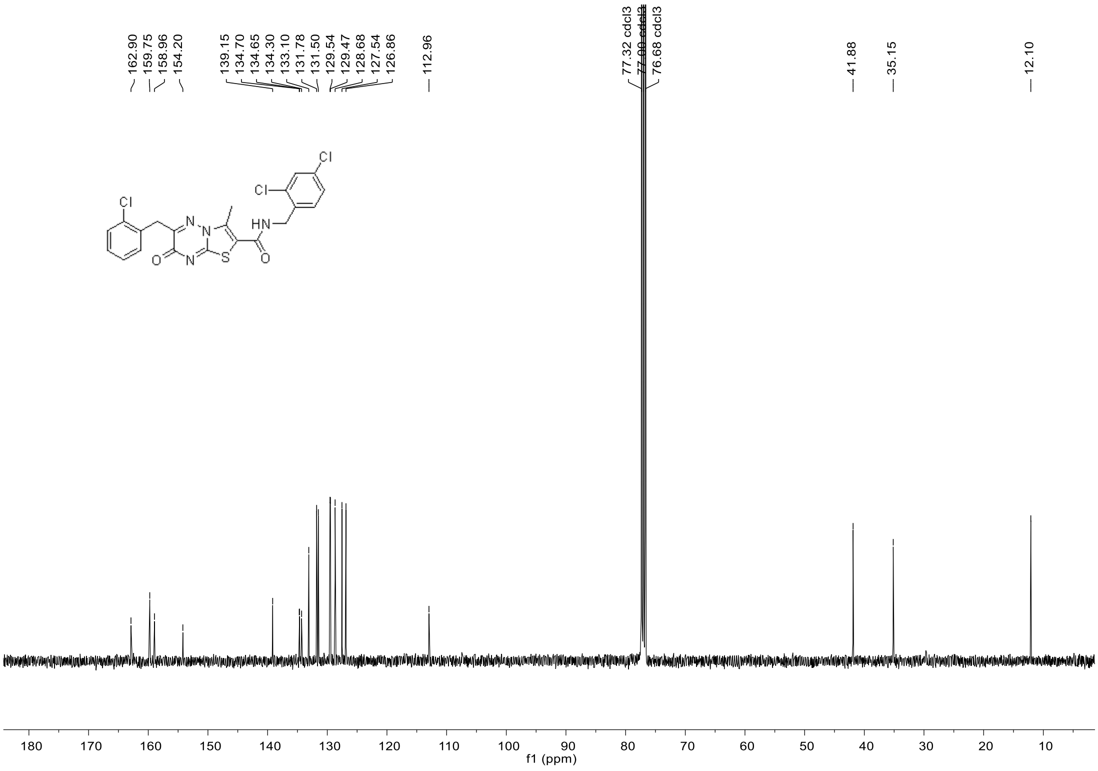

6-(2-Chlorobenzyl)-N-(4-fluorobenzyl)-3-methyl-7-oxo-7H-thiazolo[3,2-b]-1,2,4-triazine-2-carboxamide (**5d**):

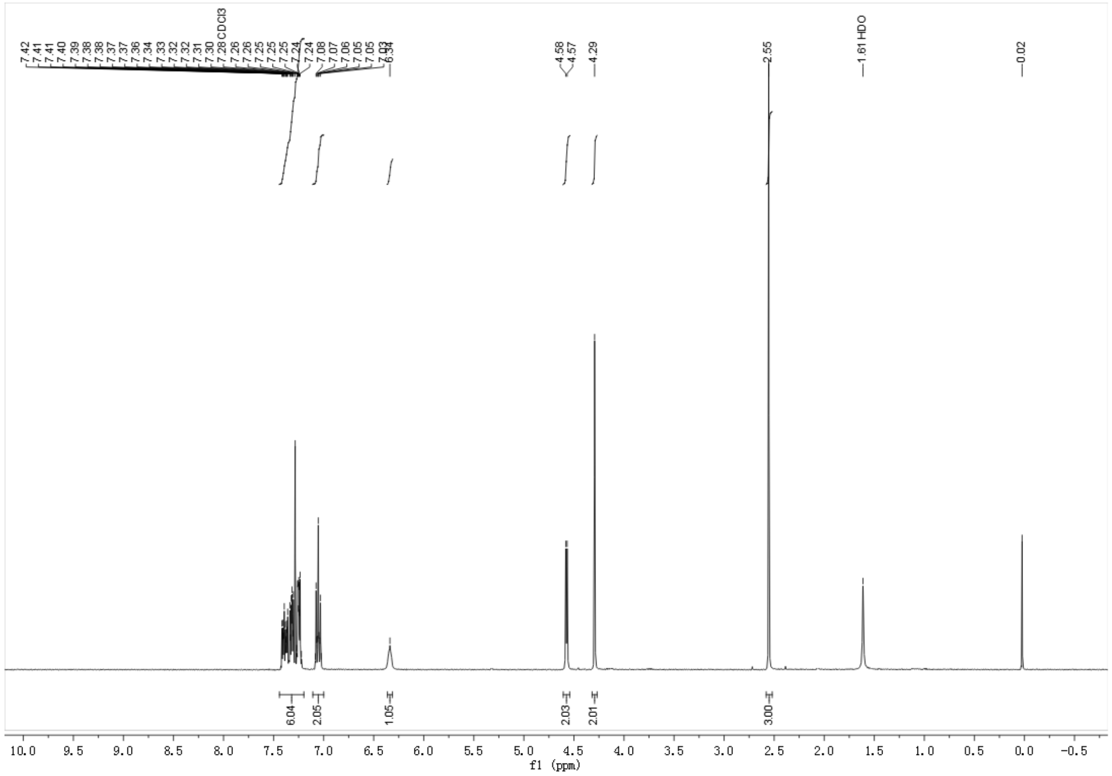

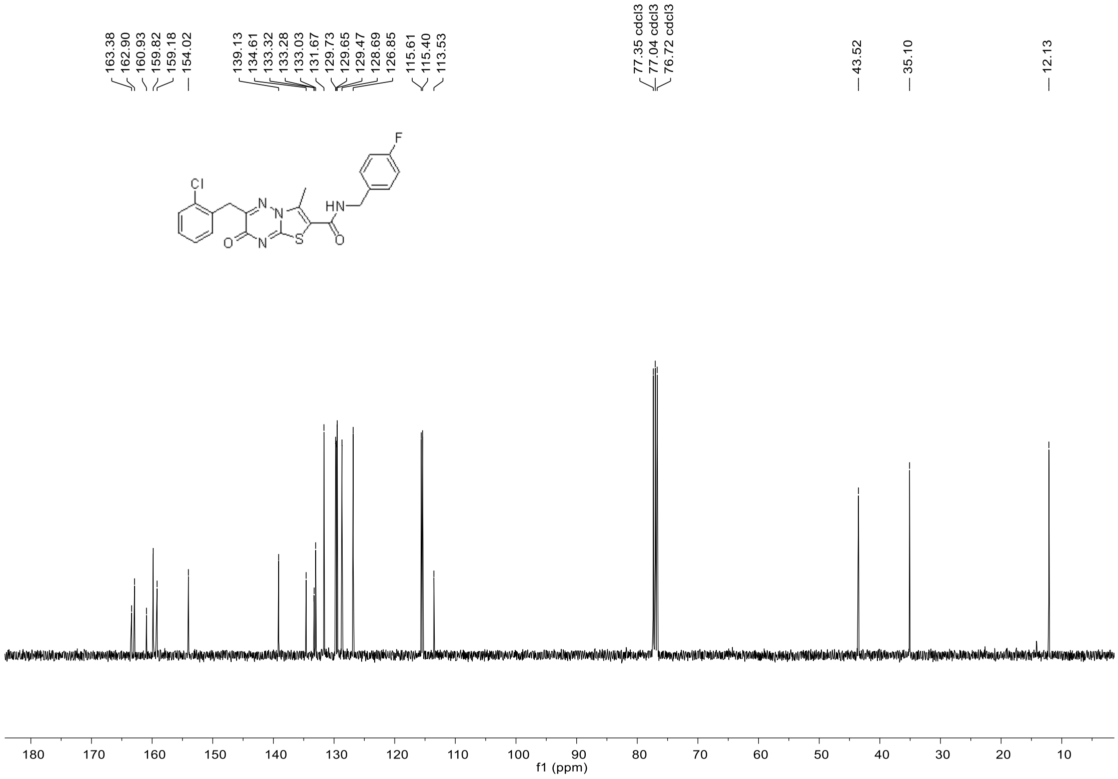

6-(2-Chlorobenzyl)-3-methyl-N-(4-methylbenzyl)-7-oxo-7H-thiazolo[3,2-b]-1,2,4-triazine-2-carboxamide (**5e**):

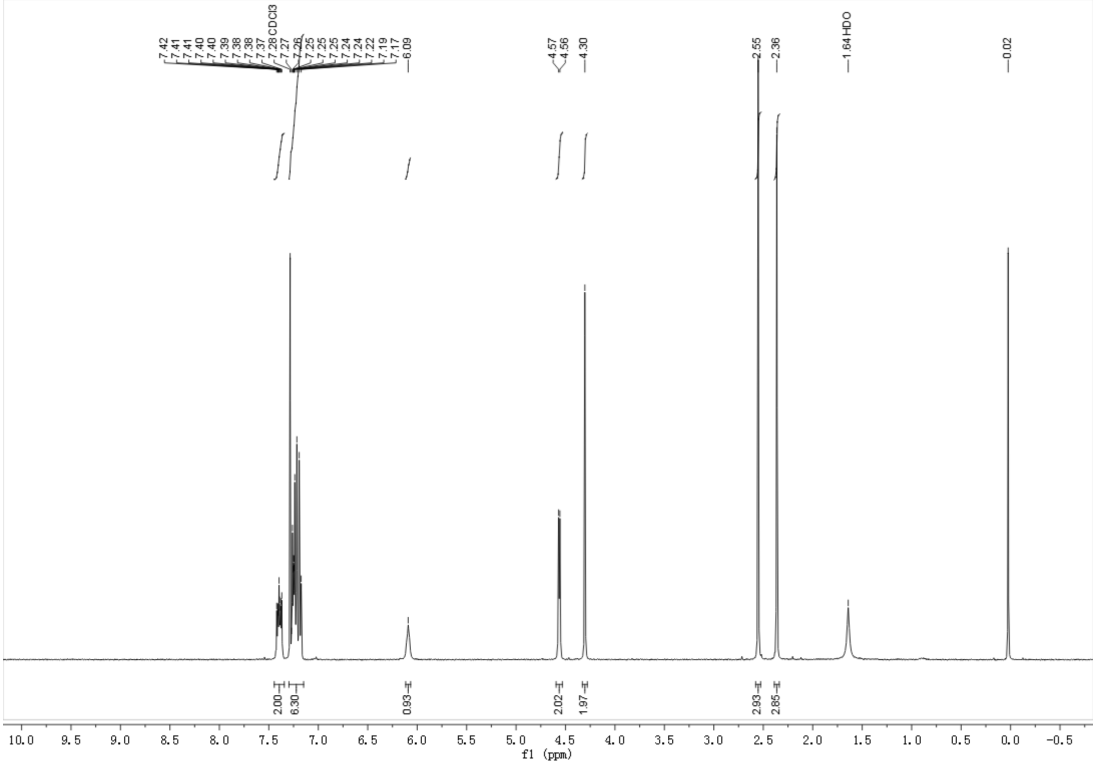

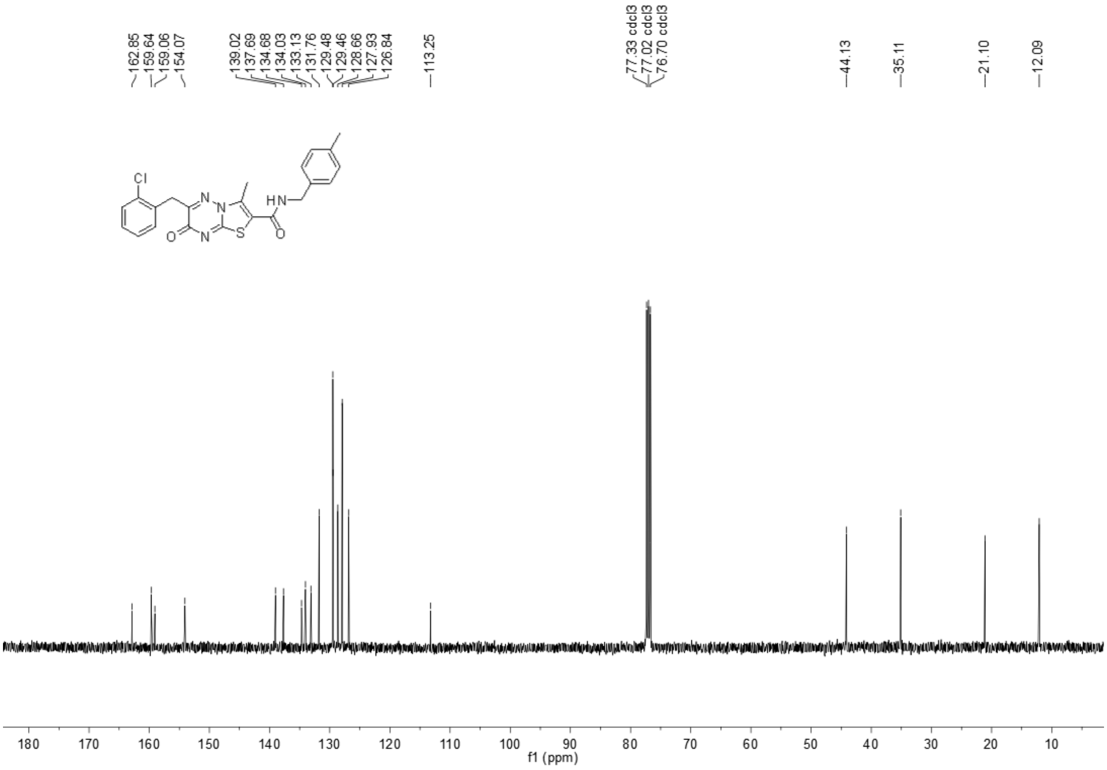

6-(4-Chlorobenzyl)-N-(4-fluorobenzyl)-3-methyl-7-oxo-7H-thiazolo[3,2-b]-1,2,4-triazine-2-carboxamide (**5f**):

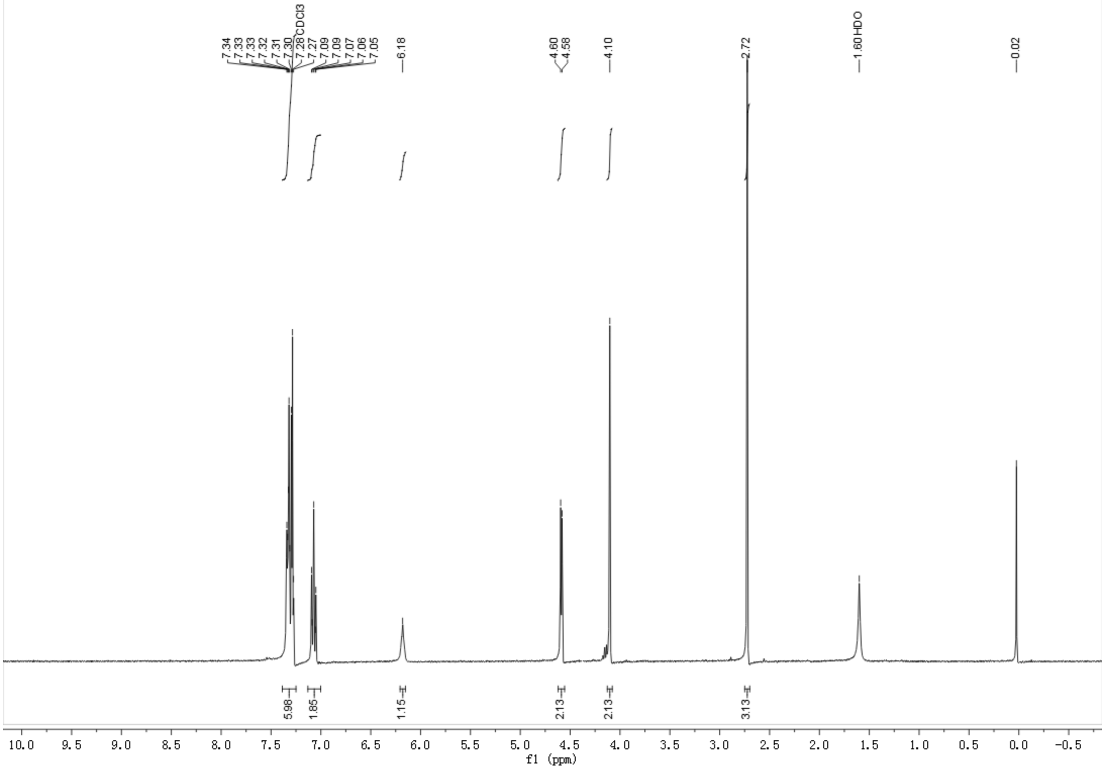

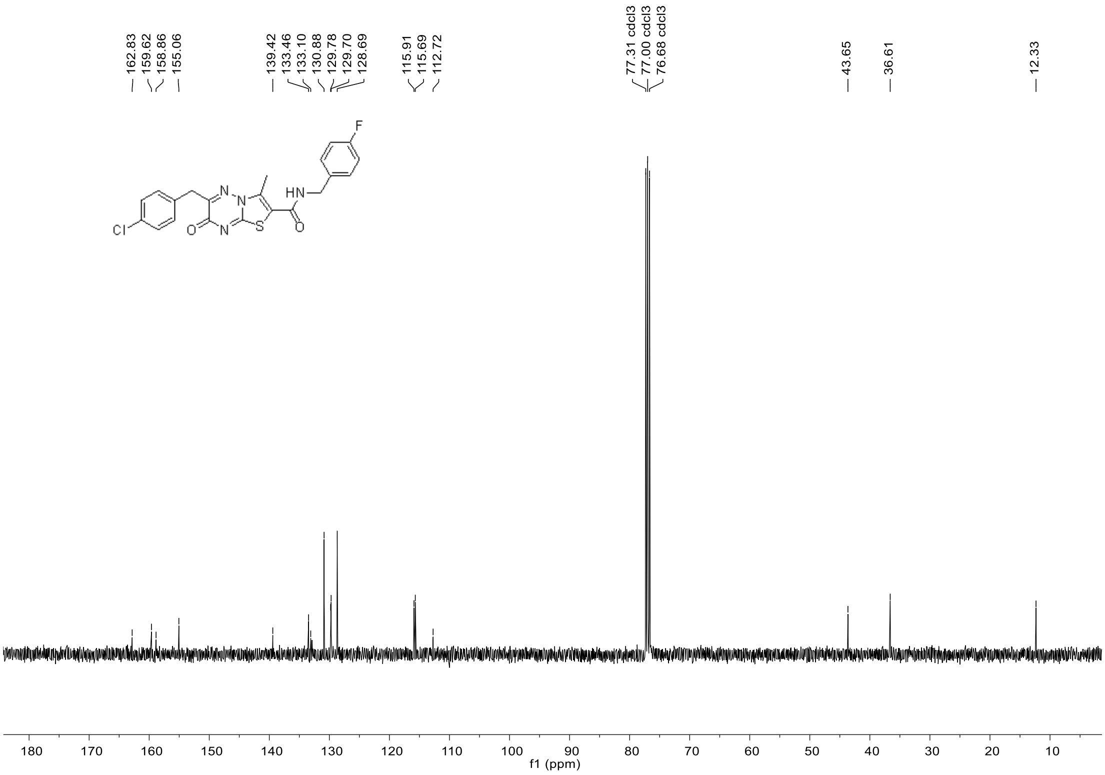

6-(4-Fluorobenzyl)-N-(furan-2-ylmethyl)-3-methyl-7-oxo-7H-thiazolo[3,2-b]-1,2,4-triazine-2-carboxamide (**5g**):

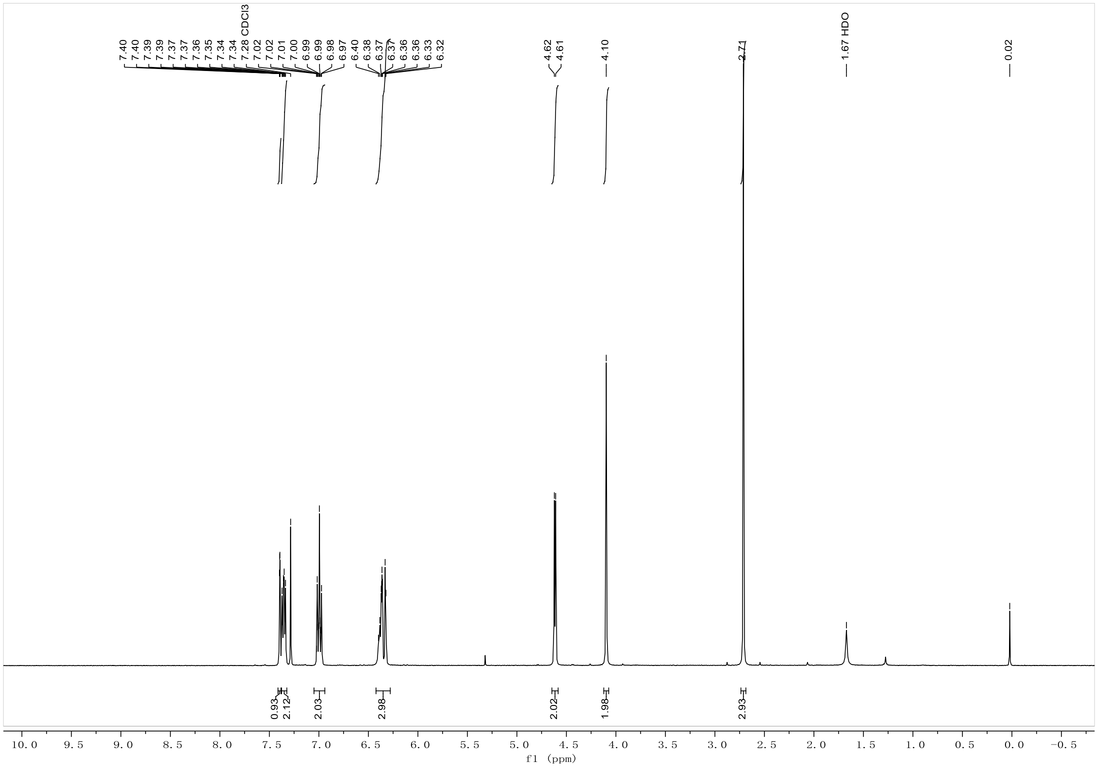

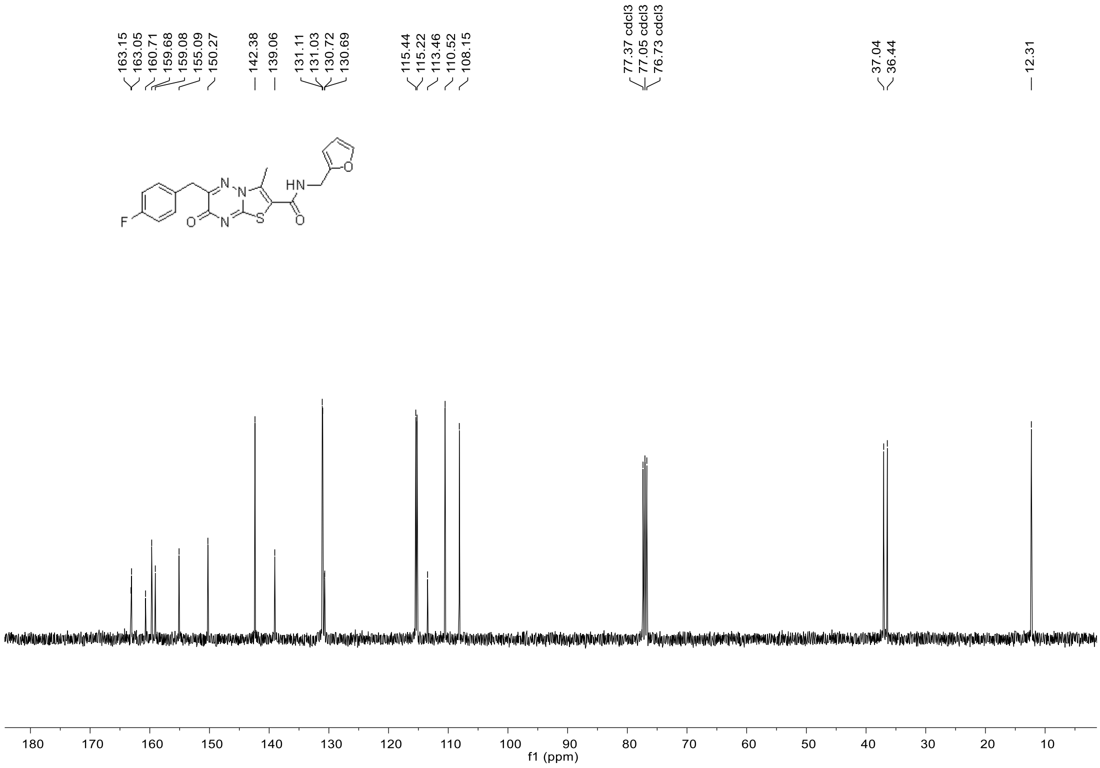

N-(2-chlorobenzyl)-6-(4-fluorobenzyl)-3-methyl-7-oxo-7H-thiazolo[3,2-b]-1,2,4-triazine-2-carboxamide (**5h**):

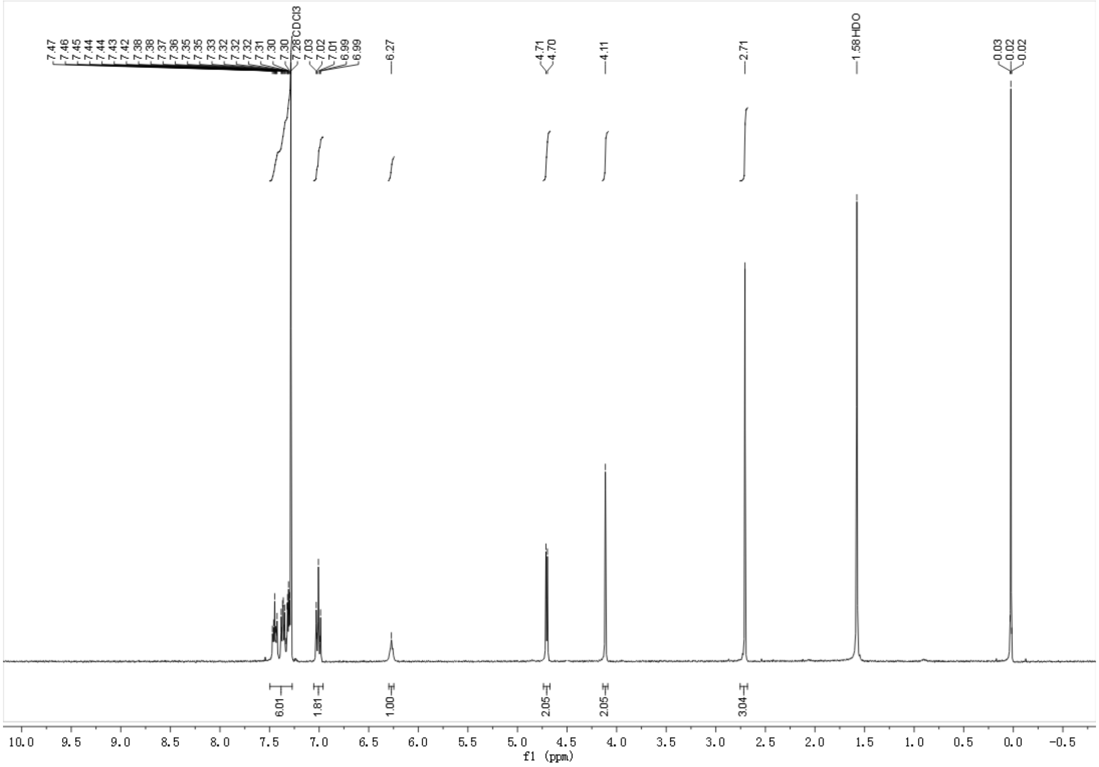

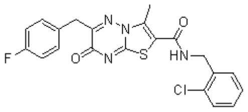

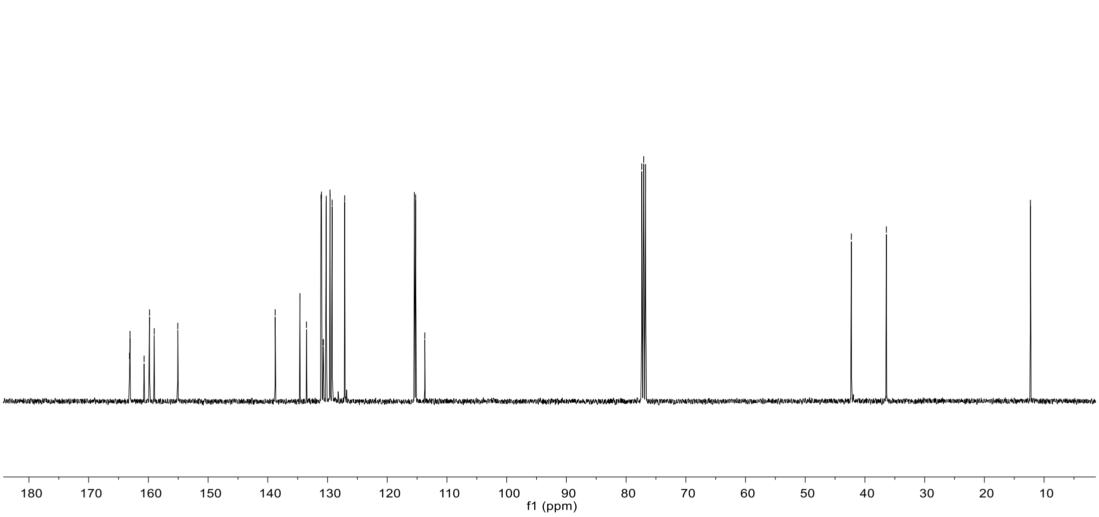

N-(furan-2-ylmethyl)-6-(4-methoxybenzyl)-3-methyl-7-oxo-7H-thiazolo[3,2-b]-1,2,4-triazine-2-carbox amide (**5i**):

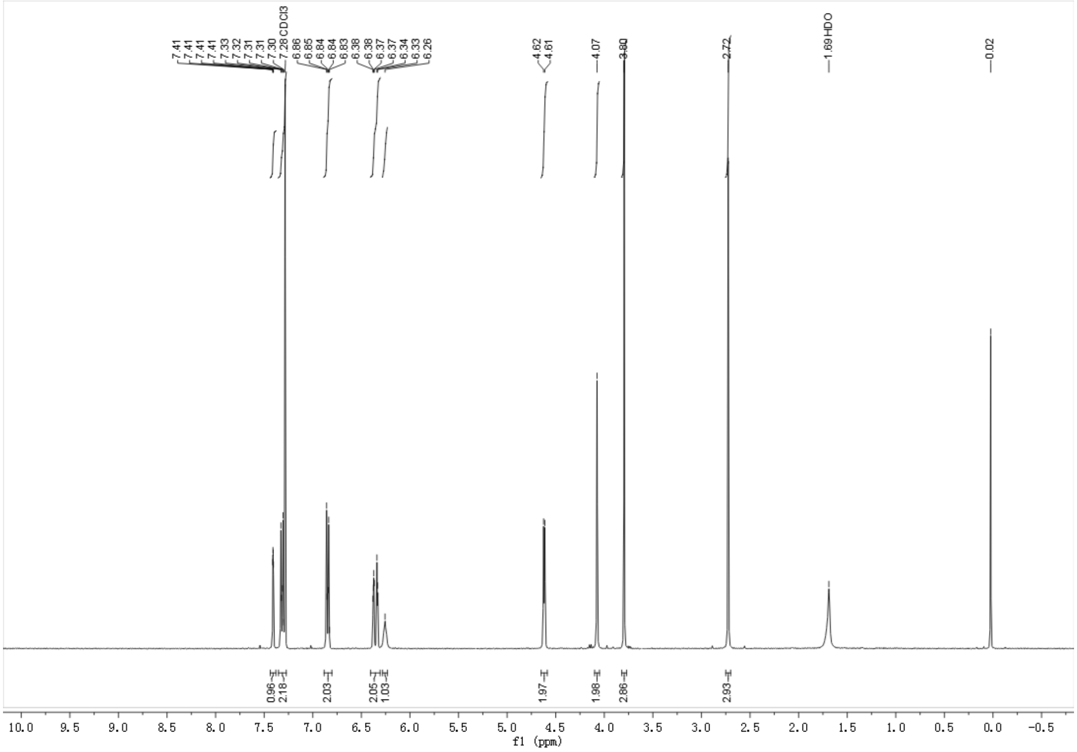

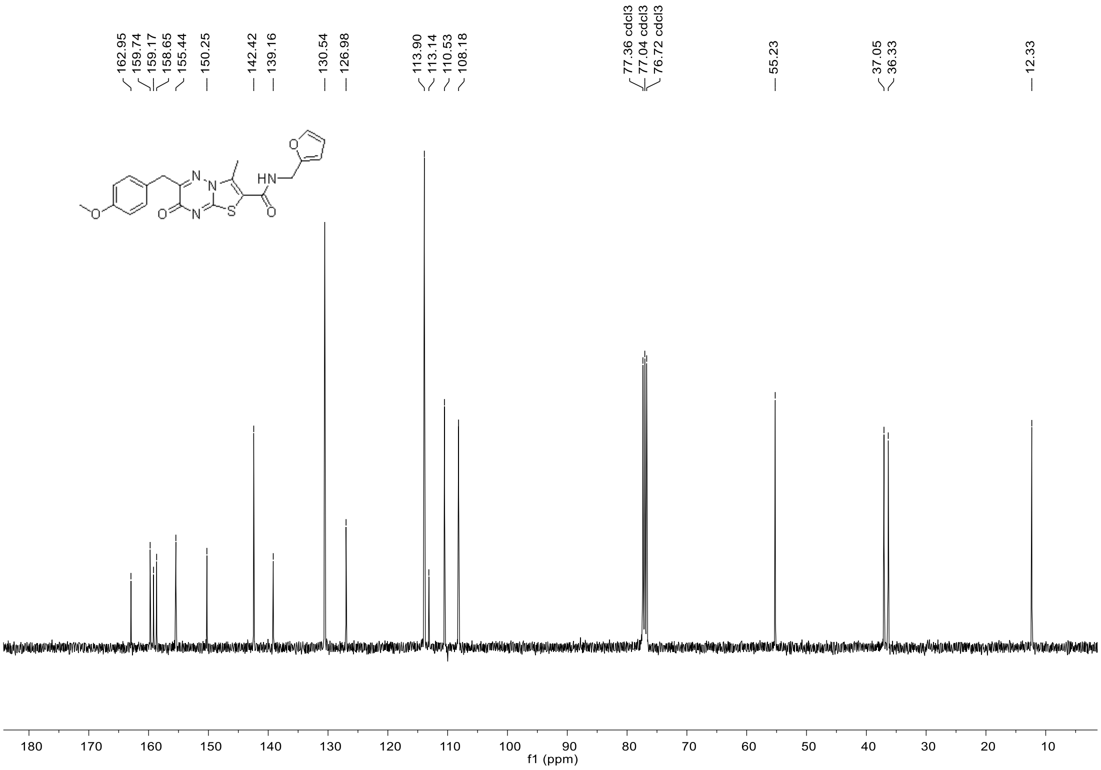

N-(2-chlorobenzyl)-6-(4-methoxybenzyl)-3-methyl-7-oxo-7H-thiazolo[3,2-b]-1,2,4-triazine-2-carboxamide (**5j**):

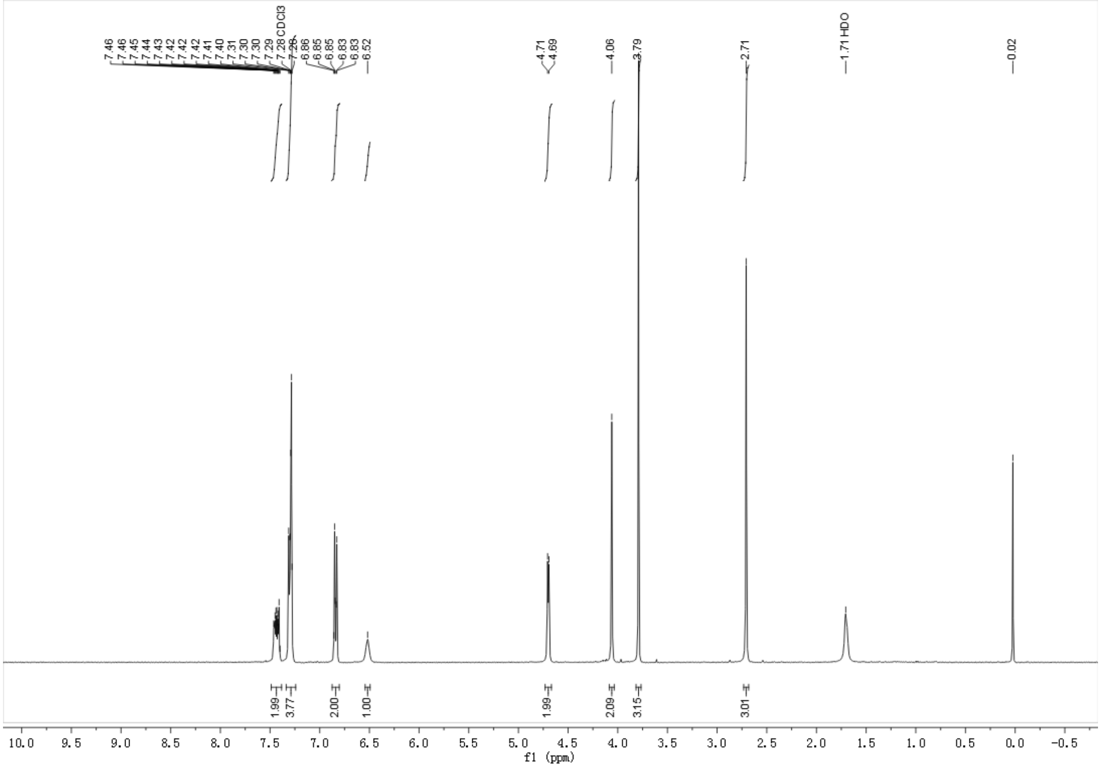

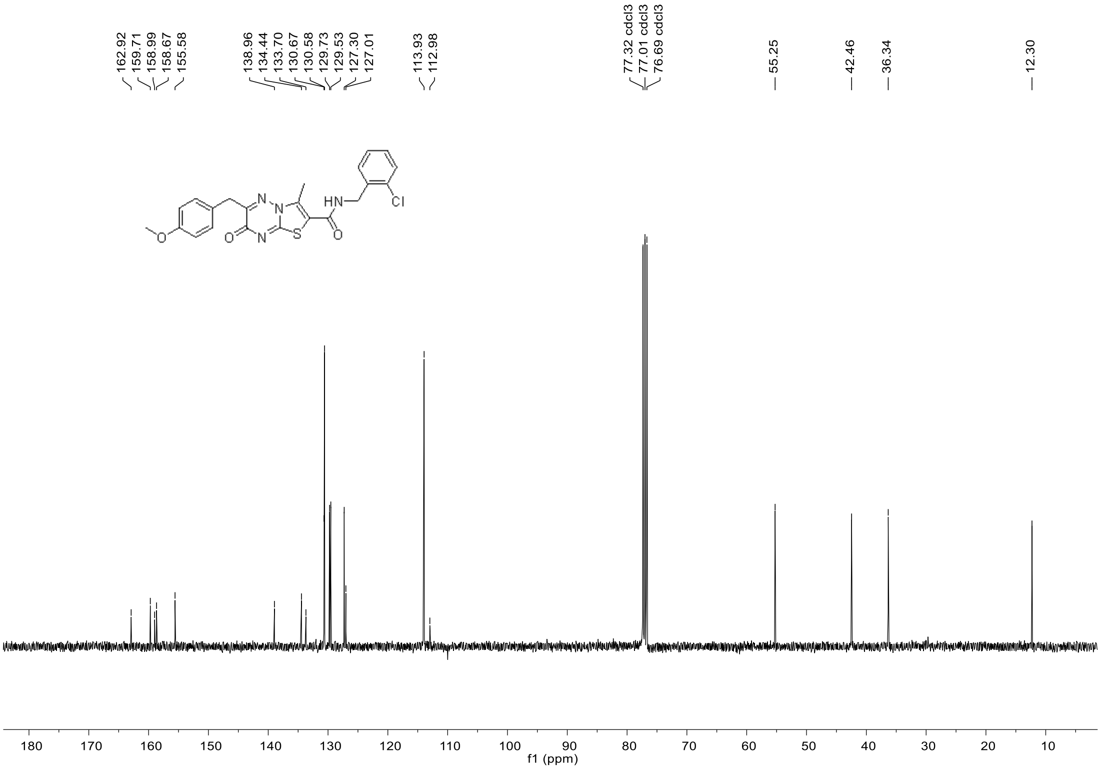

Supplement: Supplementary file 1 [file molecules-25-01307-s001.pdf]
